# Longitudinal Single-Cell RNA Sequencing Reveals Evolution of Microstates and Macrostates in Chronic Myeloid Leukemia

**DOI:** 10.1158/0008-5472.CAN-25-4371

**Published:** 2026-06-22

**Authors:** David E. Frankhouser, Dandan Zhao, Yu-Hsuan Fu, Anupam Dey, Ziang Chen, Jihyun Irizarry, Jennifer Rangel Ambriz, Tiffany Kanesa Ybarra, Sergio Branciamore, Denis O’Meally, Ryan S. Sathianathen, Jeffrey M. Trent, Stephen J. Forman, Adam L. MacLean, Ya-Huei Kuo, Kathleen M. Sakamoto, Bin Zhang, Russell C. Rockne, Guido Marcucci

**Affiliations:** 1 Department of Computational and Quantitative Medicine, Beckman Research Institute, City of Hope National Medical Center, Duarte, California.; 2 Department of Hematologic Malignancies Translational Science, Beckman Research Institute and Division of Leukemia, City of Hope National Medical Center, Duarte, California.; 3 Department of Quantitative and Computational Biology, University of Southern California, Los Angeles, California.; 4 Department of Pediatrics, Stanford University, Stanford, California.; 5 Department of Diabetes and Cancer Discovery Science, Beckman Research Institute, City of Hope National Medical Center, Duarte, California.; 6 Translational Genomics Research Institute, Phoenix, Arizona.

## Abstract

Single-cell RNA sequencing (scRNA-seq) has revolutionized our understanding of cancer. However, identifying meaningful disease states from single-cell data remains challenging due to the complex continuum of transcriptional alterations, which may obscure clear phenotype boundaries and complicate biological interpretation and clinical relevance. In this study, we systematically explored the chronic myeloid leukemia (CML)-specific information content encoded in scRNA-seq versus bulk transcriptomics to resolve this paradox and clarify how discrete disease-defining states emerge from inherently noisy single-cell data. Although CML single-cell transcriptomes existed along continuous transcriptional microstates, clinically relevant leukemia phenotypes clearly manifested only at the pseudobulk (macrostate) level. State-transition theory was leveraged to reveal how disease phenotype state transitions are governed by cell type–specific contributions. Together, these results establish a theoretical framework explaining why discrete disease phenotypes remain hidden at the single-cell scale but emerge clearly at the aggregated macrostate level, enabling previously inaccessible biological insights into leukemia evolution. By resolving how single-cell variation aggregates into macroscopic disease states, this framework provides insights into CML progression and offers a broadly applicable strategy for exploring disease dynamics across cancers and other complex conditions.

**Significance:**

Linking transcriptional single-cell microstates to emergent phenotypic macrostates provides insights into CML progression and establishes a generalizable paradigm for interpreting cancer dynamics from single-cell data.

## Introduction

Single-cell RNA sequencing (scRNA-seq) provides unprecedented detail into cellular states and heterogeneity in cancer, yet translating this detailed information into clinically meaningful chronic phase (CP) and blast crisis (BC) disease states remains challenging. A fundamental but unresolved question is how the continuous, granular information at the single-cell level relates to the discrete disease phenotypes readily observed using bulk transcriptomics. Although bulk data effectively captures disease states, single-cell analyses often yield a complex continuum of transcriptional states that may obscure clear phenotype boundaries and complicate biological interpretation and clinical relevance.

To bridge this gap, we explore the information content of single-cell compared with bulk-level transcriptomic data. We introduce a conceptual framework grounded in state-transition theory to explain an apparent paradox that emerged from our data: although individual cells exhibit continuous transcriptional variation at the single-cell level (microstates), clinical phenotypes emerge clearly only at the aggregate or pseudobulk (PsB; macrostate) level. We apply our theoretical approach to chronic myeloid leukemia (CML), a disease defined by the presence of the *BCR::ABL* fusion gene and characterized clinically by two distinct phases: initial CP and later BC. Despite successful achievement of long-term remission with tyrosine kinase inhibitors, the persistence of leukemia stem cells in CP, that could eventually drive disease transformation into BC, highlights the need for better understanding of leukemia dynamics and makes CML an ideal model system to explore how information of single cells relates to disease phenotype dynamics. To date, CML studies using scRNA-seq have primarily been used to either investigate stem and progenitor cell populations (1–3) or to identify how specific immune cells respond or influence therapy (4, 5). Here, we approach CML at the system level, and using state-transition modeling of scRNA-seq data, we quantify how the population and transcriptional changes of all peripheral blood cell types contribute information to CML development and dynamics.

## Materials and Methods

### Mouse model and experimental design

**Figure 1.**
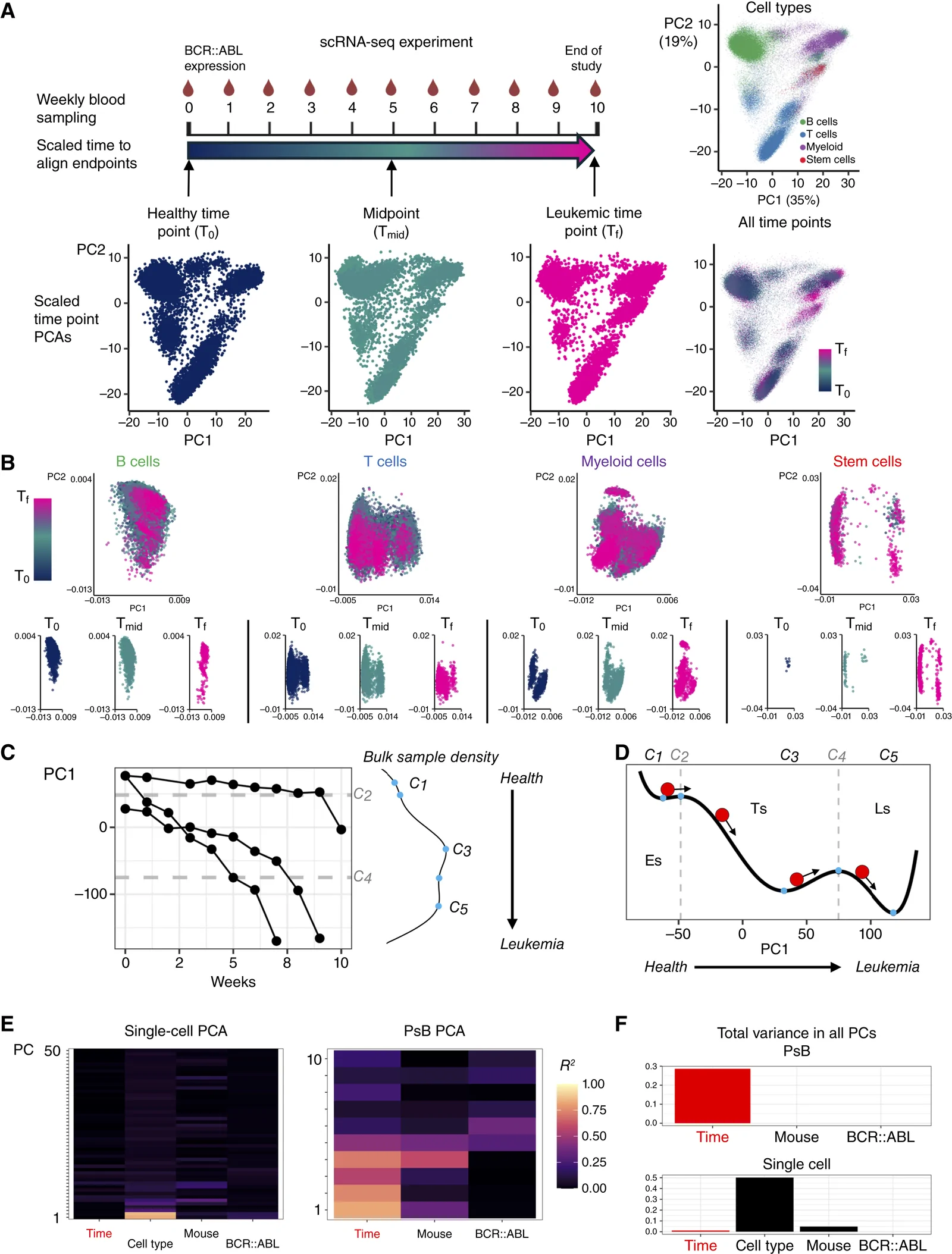
Leukemia phenotype is a macrostate observable in the pseudobulk data. **A,** Time-series PBMC samples from a CP CML mouse model were collected weekly, and scRNA-seq was performed to determine the cell contribution to leukemia state-transition (top left). Because the mice developed leukemia at different rates, a scaled time was used to align the samples from the healthy (T_0_) to leukemia (T_f_) time points. PCA was performed using all time points and all cells to determine whether a unique transcriptional state associated with leukemia (T_f_) could be identified (i.e., a SC-level CML state-space; bottom). After labeling cells by cell type and visualizing cell type in PCA space, the primary source of variance (PC1 and PC2, which accounted for 35.2% and 19.6% of the variance in the data, respectively) separated cells by cell type and not leukemic from healthy time points. **B,** PCA was performed on each cell type separately to determine whether a CML state-space could be identified within each cell type. In all PCs, the cells were observed to be in a continuum of transcriptional states that included states from both health and leukemic time points. **C,** PsB transcriptomes for each sample were created using all cells and, following the same procedure used previously to identify a CML state-space using bulk RNA-seq, a PsB CML state-space was identified in PC1 which explained 34.6% of the total variance. When plotted vs. time, each mouse’s trajectory in PC1 shows the CML development as the mice move from the healthy to leukemic state. The sample density in the bulk state-space was used to identify boundaries between stable critical points ($c_{1}$, $c_{3}$, and $c_{5}$) and the unstable boundary critical points ($c_{2}$ and $c_{4}$) in the PsB space. **D,** Using the critical points of the PsB state-space, a potential landscape was constructed that describes the sample’s CML dynamics. Each mouse is modeled as a particle undergoing Brownian motion in the potential landscape. The stable critical points are used to define the early, transition, and late phenotypic states of CML. **E,** Association between covariates and the SC- and PsB-level data were quantified using the PC structure produced by each data type. Partial regression, performed for each covariate while adjusting for all others, was used to produce *R*^2^ values for each PC. Time was used as a proxy for CML evolution as the mice developed leukemia over the course of the time-series sampling. In PsB data, time showed strong association with the leading PCs which capture the dominant sources of variation, whereas in the SC-data, time exhibited uniformly weak associations across PCs. **F,** Variance attributable to each covariate was summarized by weighting partial *R*^2^ by each PC’s variance fraction and summing across PCs. Time dominates variation in PsB but contributes little in SC data.

The SCLtTA/BCR::ABL and *Mir142*^−/−^SCLtTA/BCR::ABL mice in B6 background were maintained on tetracycline (tet) water at 0.5 g/L. Tet withdrawal (tet-off) results in endogenous expression of *BCR::ABL* and generation of a CP CML-like disease in SCLtTA/BCR::ABL mice with a median survival of approximately 55 days and of a BC CML-like disease in *Mir142*^−/−^SCLtTA/BCR::ABL mice with a median survival of approximately 32 days, after induction of *BCR::ABL*. Blood (100 μL) was collected from 6- to 8-week-old male (*n* = 1) and female (*n* = 2) SCLtTA/BCR::ABL or *Mir142*^−/−^SCLtTA/BCR::ABL mice weekly, i.e., before *BCR::ABL* induction (*t* = 0) and after induction up to 9 weeks (*t* = 1–9) or when the mouse developed leukemia and became moribund, whichever event occurred first (Fig. 1A). After red cell lysis, an aliquot of the peripheral blood mononuclear cells (PBMC) was stained with anti-mouse antibodies and analyzed for hematopoietic cell subpopulations, and the remaining cells were subjected to scRNA-seq. All animal studies were performed in accordance with federal guidelines and protocols and were approved by the Institutional Animal Care and Use Committee (IACUC) at City of Hope under IACUC protocol 15005.

### Sequencing and data processing

Approximately 10,000 cells per murine sample were captured using a Chromium X (10X Genomics), using the Chromium Next GEM Single Cell 3′ Reagent Kit v3.1 (10X Genomics, cat. #PN-1000268). Prior to sequencing, library quality was assessed using a High-Sensitivity DNA Chip (Agilent, cat. #5067-4626) and quantified using the Qubit High-Sensitivity DNA Assay Kit (Thermo Fisher Scientific, cat. #Q32854). Sequencing was performed on a NovaSeq 6000 (Illumina) using the S4 Reagent Kit v1.5, 200 cycles (part no 20028313). Illumina Real-Time Analysis (RTA3) v3.4.4 was used for base calling and quality scores, and Illumina BCL Convert v3.9.3 to export FASTQ files. All protocols were performed according to the manufacturer’s instructions. Comprehensive library quality assessment was performed using v2.1.0 of the nf-core/scrnaseq pipeline (6), and reads (including those from introns) counted using 10X Genomics Cellranger count v7.0.0 implemented therein. We used the Gencode M28 reference annotation and genome (https://www.gencodegenes.org/, downloaded March 22, 2022) augmented with a custom *BCR::ABL* transcript (GenBank accession forthcoming) prepared using the default parameters and preprocessing described for Cellranger mkref. To perform quality control, each library was filtered to remove genes expressed in fewer than three cells and cells with fewer than 200 genes expressed, more than 20% mitochondrial transcripts, or fewer than 5% ribosomal transcripts. The filtered libraries were then merged into a single v5 Seurat (7) object for downstream analysis. No formal batch integration or correction was applied. Cell types were assigned with the default parameters of SingleR (v2.6.0; ref. 8) using reference expression profiles from the Immunological Genome Project, accessed through the celldex package (v0.1.16.0; Supplementary Table S1). Raw sequence reads and the Seurat object are available via the Gene Expression Omnibus (GEO; GSE296507). Differentially expressed genes (DEG) were identified using the Seurat FindMarkers function with default parameters testing all genes that were expressed in at least 10% of the cells included in the comparison.

### State-space exploration at the SC level

Like the previously described bulk RNA-seq CML state-space (9) which separated health from leukemic transcriptional state and defined continuous trajectories for our time-series samples, the scRNA-seq data were investigated for a similar CML state-space (Supplementary Fig. S1A). Here the goal was to find a lower dimensional representation of the data that captured the progression of a sample from health to leukemia in either all cells or a subset of cell types. We expected an SC-level CML state-space to have similar properties such that the healthy (T_0_) cells and the leukemic (T_f_) cells would occupy distinct transcriptional states defined by a lower dimensional representation of the data. Different dimensionality reduction techniques were applied to the SC-level data using all cells and all samples as input (Fig. 1A; Supplementary Fig. S1B–S1F). Principal component analysis (PCA) was performed using the Seurat package on the log-normalized mean-centered gene expression (7). Based on the elbow of the scree plot of the singular values (Supplementary Fig. S1G), the first six principal components (PC) were assessed for cells occupying unique transcriptional state associated with the healthy (T_0_) and leukemic (T_f_) states (Fig. 1A; Supplementary Fig. S1B). Uniform Manifold Approximation and Projection for Dimension Reduction (UMAP), diffusion map, and two scRNA-seq analysis tools, Decipher and Mellon, were also used to try to identify a CML state-space (Supplementary Fig. S1C, S1E and S1F; refs. 10, 11). Mellon was used with default parameters to identify cell clusters that changed density over leukemia development but did not reveal any cell clusters that were uniquely associated with disease state (Supplementary Fig. S1F). Because Decipher was able to construct a latent space where divergent AML trajectories between sorted cells from healthy and leukemic bone marrow (BM) samples (11), we applied it to our more diverse PBMC CML samples. To train the Decipher model, healthy time points (T_0_) were used as controls and leukemic time points (T_f_) were used as leukemic samples. Decipher was applied to all cells and each of the four cell types to try to identify cell type–specific disease trajectories; however, none of the Decipher latent spaces constructed trajectories that described leukemic development in our CML mice (Supplementary Fig. S1E).

Each cell type was also analyzed separately to determine whether an SC-level state-space could be identified (Fig. 1B; Supplementary Fig. S1D). For each cell type, singular value decomposition (SVD) was performed on the log-normalized mean-centered expression of all cells and visualized to try to identify any PC or combination of PCs where the cells from the leukemic time points occupied a unique transcriptional state. Despite each cell type showing DEGs between T_0_ and T_f_ time points (Supplementary Fig. S1H), no obvious T_0_ and T_f_ states were observed. This analysis was repeated for the BC CML mice.

### PsB state-space construction

The PsB data were constructed by summing gene counts within each mouse’s time point samples. For each sample, the counts were normalized to counts per million (CPM). This resulted in a sample by gene expression (*n* = 29,513) matrix similar to what would have been obtained if bulk RNA-seq had been performed on each PBMC sample. SVD was performed on the mean-centered, log_2_ normalized PsB CPM which decomposes the gene expression matrix ($X=U\Sigma V^{*}$) into the following: U, which provides components of the data in decreasing total variance explained (i.e., the PC), Σ, which are the eigenvalues of each PC, and $V^{*}$, which are the loading values for how the genes are combined to construct each PC (Supplementary Fig. S2A). The top six PCs were visually inspected using scaled time to identify whether they encoded mouse trajectories that separated healthy (T_0_) from leukemic (T_f_) time points, and all PCs were correlated with the *BCR::ABL* expression in each PsB sample (Supplementary Fig. S2B; Supplementary Table S2). The *BCR::ABL* expression was determined by aligning the scRNA-seq reads to the BCR::ABL fusion gene transcript, normalizing based on the total aligned reads per each cell and then summing the cell level reads to get the PsB *BCR::ABL* expression.

### CML potential

The PsB state-space was compared with the bulk state-space by identifying healthy and leukemic reference or “anchor” points and then scaling the bulk state-space to span the PsB state-space. For the bulk state-space, the healthy anchor point was the mean bulk state-space coordinate of all week 0 samples, and the leukemic anchor was the mean bulk state-space coordinate of final time point for all *BCR::ABL* induced mice that developed leukemia. For the PsB state-space, the healthy anchor point was the mean PsB state-space coordinate of all the week 0 samples, and the leukemia anchor point was the mean PsB state-space coordinate of final time point for the mice that developed leukemia. Using these anchor points, the bulk state-space coordinates of all samples and the location of the state-space critical points were translated into the PsB state-space (Supplementary Fig. S2C). We observed that the bulk and PsB trajectories described similar dynamics in the combined state-space. Although the PsB mice developed leukemia quicker, this was expected as the mice used in this experiment were homozygous *BCR::ABL* knock-in, whereas the mice used in the bulk RNA-seq experiment were heterozygous for *BCR::ABL* and consequently would receive reduced oncogene expression upon induction. Given the similar dynamics and higher density of samples in the bulk state-space, we used the translated bulk state-space critical points (Fig. 1C) and resulting potential (Fig. 1D) for the PsB which was constructed as described previously (9).

### Variance attributable to covariance in SC- and PsB-level data

Both the marginal and the partial *R*^2^ values were multiplied by the variance explained by each corresponding PC and then summed over all PCs to estimate the total variance attributable to each covariate in each of the SC, PsB, and cell type SC-level data (Fig. 1E and F; Supplementary Fig. S3A and S3B).

### Marginal and partial associations with each PC

For each PC, association with each covariate was quantified using both marginal and partial regression models. Marginal association was computed as the *R*^2^ from a univariate linear model in which each PC was a function of each variable (PC ∼X). Partial association was computed as the incremental variance explained by adding a covariate X to a base model containing the remaining covariates, using the partial *R*^*2*^ definition $R_{\partial }^{2}\left(X\right)=\frac{SSE_{reduced}-SSE_{full}}{SSE_{reduced}}$, in which $SSE_{reduced}$ denotes the residual sum of squares of the reduced model (excluding X) and $SSE_{full}$ denotes the residual sum of squares of the full model (including X). This procedure was repeated for each covariate and each PC of both the PsB- (Fig. 1E; Supplementary Fig. S3A) and SC-level (Supplementary Fig. S3B) data.

### Variance-weighted summaries across PCs

To summarize how much variance in the representation was aligned with each covariate, per-PC *R*^2^ values were combined across PCs using variance weighting $Vr\left(X\right)=\overset{K}{\underset{k=1}{\sum }}var_{k}\cdot R_{k}^{2}\left(X\right)$, in which ${var}_{k}$ denotes the fraction of variance explained by ${PC}_{k}$ and K is the number of PCs included (*K* = 50 for SC-level, *K* = 10 for PsB, and *K* = 10 for each isolated cell type SC-level data). Variance-weighted summaries were computed separately for marginal and partial *R*^2^ (Fig. 1F; Supplementary Fig. S3A).

### SVD and projection cell type pseudobulk samples into the state-space

**Figure 2.**
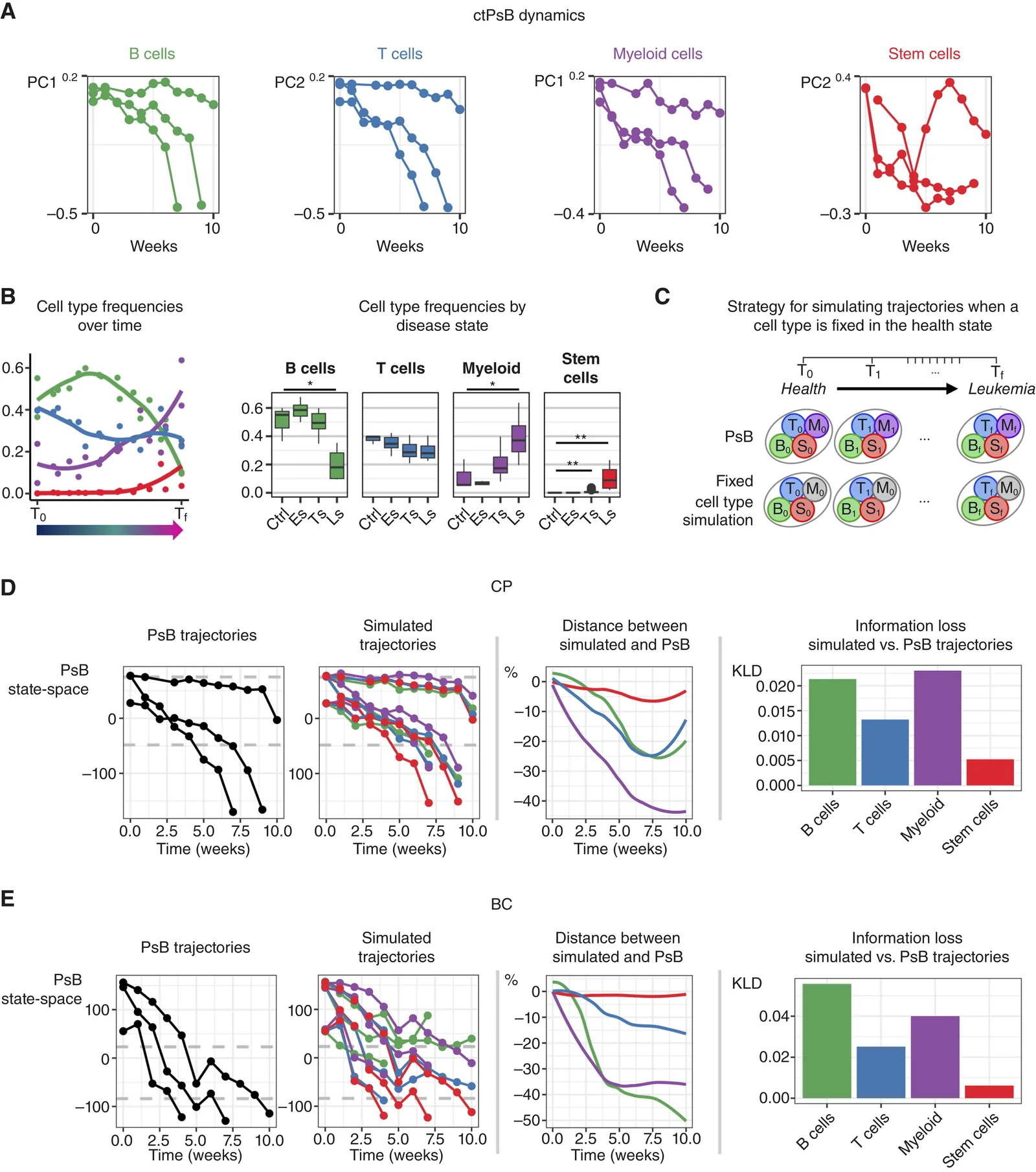
Cell types contribute different information to leukemia dynamics. **A,** Cell type PsB transcriptomes were created for each sample by combining gene counts from the cells of each cell type. Using the same procedure to identify the PsB state-space, a CML state-space was identified in either PC1 or PC2 for each cell type. The ctPsB state-spaces showed different leukemia development dynamics when compared with the PsB state-space. **B,** The number of cells in each cell type is shown as a proportion over the scaled time of leukemia development (left). The cell type proportions are grouped by disease state to test cell type population changes over leukemia development (*, *P* < 0.05; **, *P* £0.001). **C,** Computational simulation strategy to quantify each cell type’s contribution to CML dynamics is depicted in the diagram. Each cell type was fixed at the healthy (T_0_) time point by replacing all cells of that type with the healthy time point cells for all subsequent time points. Those cells were then used to construct a simulated PsB sample for each time point sample to simulate the effect of each cell type being fixed at T_0_ cell population count and gene expression state. **D,** Full PsB trajectories for the CP CML mice were compared with the simulated trajectories where each cell type is fixed at the healthy (T_0_) time point (left). For each sample, the distance between the full and simulated trajectories was calculated as a percentage and then summarized by each cell type to show how much less state distance the fixed cell type simulations traveled in the state-space (middle). To quantify the information loss from fixing each cell type, KLD was calculated between the full PsB and simulated trajectories for each cell type to show that myeloid cells contributed the most information to the state-transition if not allowed to evolve. **E,** The same simulations were performed for the BC CML mice which showed different contribution from the cell types with B cells contributing the most information to BC CML state-transition. KLD, Kullback–Leibler divergence.

For each cell type, a cell type PsB sample was constructed for each time point sample. The cell type pseudobulk (ctPsB) was constructed using the same data and method as the PsB samples, except only the cells of each cell type were included. For each cell type, the ctPsB data were mean-centered and log_2_-normalized before performing SVD (Supplementary Fig. S2A). The top PCs were investigated for scaled time and other descriptive variables to explain the top sources of variance in each cell type. The ctPsB CPM values were also used to project each ctPsB into the PsB state-space. First, the ctPsB data were mean-centered using the means from the PsB data, and then they were log_2_-normalized. To project the data into the PsB state-space, the PsB PCs of the ctPsB were calculated as $U_{ctPsB}=X_{ctPsB}V\Sigma^{-1}$, in which $X_{ctPsB}$ are the mean-centered log_2_ ctPsB expression matrix and V and $\Sigma^{-1}$ are the loading values and singular values from the PsB SVD results, respectively (Supplementary Fig. S2A). For both the ctPsB SVD results (Fig. 2A; Supplementary Fig. S3C) and the ctPsB projections (Supplementary Fig. S3E), PC1, which corresponded to the PsB state-space, was plotted with the PsB state-space versus time for each cell type separately to observe the location and dynamics each ctPsB had in the PsB state-space. The critical points for each set of ctPsB trajectories and the resulting potentials were estimated by observing the ctPsB trajectory dynamics in the ctPsB state-spaces (Supplementary Fig. S3D) and when projected into the PsB state-space (Supplementary Fig. S3E). For the ctPsB projections, the location and the distance that each set of trajectories overlapped the PsB trajectories was summarized using the maximum and minimum values observed for each cell type to create a spanning vector (Supplementary Fig. S3F). To explore how *BCR::ABL* expressing cells related to cell type, we plotted *BCR::ABL*-positive cells in the PCA space and found the fraction of cells positive for *BCR::ABL* in each cell type (Supplementary Fig. S3G). The same processing was applied to BC CML samples to explore whether an SC-level state-space could be constructed, to construct the BC PsB state-space, and to construct and project the ctPsB state-space (Supplementary Fig. S4A–S4G).

### Cell type population changes

The number of each of the four cell types identified in each sample was used to calculate the proportional representation of each cell type and plotted over scaled time (Fig. 2B; Supplementary Table S3). The samples were then grouped by disease states, as defined by the critical points from the PsB state-space and *propeller* from the *speckle* package in R (12). A false discovery rate *P* value <0.05 was used to determine significant changes in cell type populations.

### Fixed cell type computational simulation

The contribution of each cell type to the state-space trajectories was assessed using a computational simulation strategy. The simulation was performed separately for each cell type. For the cell type being simulated, all cells identified as that cell type were removed from each time point sample. Then, the gene expression for all the week 0 cells identified as that cell type were added to each time point sample (Fig. 2C). The result for each mouse was that one cell type was fixed to the week 0 cell count and transcriptional state while all other cell types underwent CML development. Next, the fixed simulation for each time point sample was aggregated into PsB data. The PsB data were then projected into the PsB state-space so that the fixed simulation trajectories for each cell type could be compared with the observed PsB trajectories for each mouse (Fig. 2D). The percent distance between each cell type’s fixed simulation and the observed PsB trajectory was calculated by first taking the difference between the observed and the fixed simulation PsB state-space coordinates for each time point sample. Those differences were then divided by the maximum distance spanned by each mouse’s observed trajectory which was the difference between the maximum and the minimum PsB state-space coordinate for each mouse. Finally, a fit line was drawn for the resulting percent differences of each cell type and plotted as a function of time [Fig. 2D (middle)]. The difference in information contained in the trajectories of each fixed cell type simulation versus the observed trajectories was calculated using the Kullback–Leibler divergence which takes larger values when the simulated trajectories encode less of the observed trajectories [Fig. 2D (right)].

### Application of state-transition to BC samples

The BC CML data were collected using an identical time-series experiment as the CP CML mice. The BC CML scRNA-seq samples were processed identically, and all analyses were conducted as described in the CP CML mice. The PsB state-space was determined by performing SVD on the PsB gene expression matrix, which also identified PC1 as the PsB state-space for BC CML (Supplementary Fig. S4D). As the BC CML genotype differed from the CP CML genotype used in both the scRNA-seq and bulk RNA experiments, PsB state-space dynamics and resulting potential were determined using only the BC CML mice. By assessing the sample distribution in the PsB state-space using both histograms and kernel density, the BC CML state-space was determined to have two stable critical points and one unstable critical point (Supplementary Fig. S4D). Using the same approach applied to the CP CML mice, the ctPsB state-spaces were identified to be encoded in PC1 for each cell type (Supplementary Fig. S4F). When the ctPsB data were projected into the PsB state-space, the ctPsB trajectories were shown to have a different location and contribution to the PsB state-space (Supplementary Fig. S4G). Using the BC CML PsB state-space, the fixed cell type simulation strategy was applied to the BC CML mice to quantify the information lost when each cell type was fixed (Fig. 2E).

### Human CD34^+^ BM samples and analysis

Clinical and demographic features at diagnosis were extracted from electronic medical records or provided by the Children’s Oncology Group (COG) for pediatric patients (<18 years) with CML. BM samples from patients with CML were collected through voluntary participation in existing tissue banking studies conducted at the Stanford University School of Medicine and the COG, in compliance with the institutional review boards. Written informed consent for the tissue banking studies was obtained from all participants in accordance with the Declaration of Helsinki.

**Figure 3.**
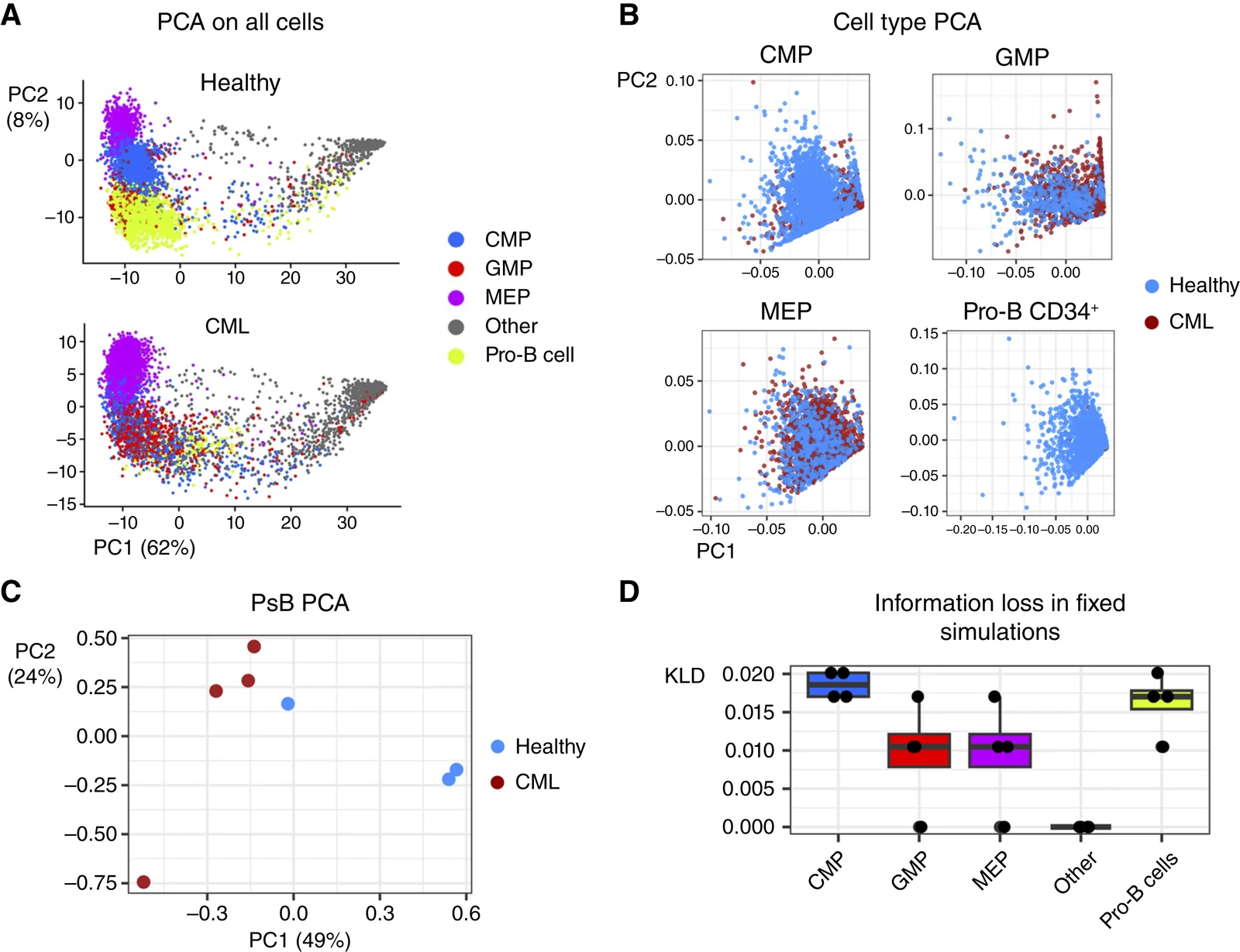
Validation on human CD34^+^ BM cells. **A,** Using a cohort of four CML and three healthy BM samples, CD34^+^ cells were isolated, and scRNA-seq was performed. Cell types were labeled from the data, and PCA was performed to determine whether a unique transcription disease could be identified from the data. However, similar to the mouse model, cell type and not the disease status of the samples was the primary source of variance. **B,** PCA was also performed on each cell type separately to identify whether disease state emerged at the single-cell level, but the healthy and CML cells were observed to be in a continuum of transcriptional states in all PCs. **C,** PsB samples were made for all cells in each sample, and PCA was performed. When PC1 vs. PC2 were plotted, PC1 separated CML from healthy samples (percent of total variance explained reported below each axis label). **D,** Fixed simulations were used on all pairwise combinations of the healthy and CML samples to quantify how each cell type contributed to CML. For each pairwise healthy–CML sample pairing, the KLD was calculated to quantify how much information was lost for each cell type and the mean from all pairwise pairings was reported. The CMP contributed the most information whereas the “other” cell type group that combined all remaining cell types accounting for less than 5% of the total cells in both healthy and CML samples contributed almost no information to CML. CMP, common myeloid progenitor; KLD, Kullback–Leibler divergence.

Cryopreserved human healthy pediatric CD34^+^ BM samples were commercially purchased from STEMCELL Technologies (cat. #7000.2) and Lonza Bioscience (cat. #2M-101). Cryopreserved BM aspirates from pediatric patients with CML BM at diagnosis were obtained from the Stanford Bass Center Tissue Bank. Lineage-/CD34^+^ cells were isolated by fluorescence-activated cell sorting for the four pediatric CML BM samples. All samples were processed by collaborating with the Stanford Functional Genomics Facility. The Chromium Next GEM Single Cell 3′ Reagent Kit (10x Genomics; cat. #PN-1000268) was used for single-cell capture and library preparation. Captured cell counts varied across samples, as healthy samples contained a median of ∼6,200 cells (range, 3,200–8,300), whereas CML samples contained a median of ∼6,100 cells (range, 800–11,500). The libraries were sequenced on a NovaSeq 6000 (Illumina). Reads were counted using 10x Genomics Cellranger count v6.0.0 with the 10x GRCh38-2020-A reference transcriptome. Libraries were then aggregated with Cellranger aggr v6.0.0 prior to being combined into a single v4 Seurat object. To perform quality control, the Seurat object was filtered to remove genes expressed in fewer than three cells and remove cells with fewer than 200 genes expressed, greater than 8,000 genes expressed, or more than 10% mitochondrial transcripts. Cell types were assigned with the default parameters of SingleR (v2.6.0; ref. 8) using reference expression profiles from the Human Primary Cell Atlas, accessed through the celldex package (v0.1.16.0; Supplementary Fig. S5A). PCA and UMAP were performed for all cells and for cells of each cell type as previously described to try to identify emergent cell states (Fig. 3A and B; Supplementary Fig. S5B and S5C). For each sample, a PsB sample was constructed and SVD was performed on all PsB samples to identify the CML state-space in PC1. Fixed cell type simulations were performed as described previously, except to accommodate the unpaired samples in this dataset, each CML sample (*n* = 4) was paired with a healthy sample (*n* = 3), and all possible pairings of CML and healthy samples were performed by performing the fixed cell type simulation on each of four possible groupings (Supplementary Fig. S5D). The information lost from each simulation was calculated by comparing the full and simulated trajectories for each sample pairing using Kullback–Leibler divergence as previously described, and the mean divergence across the four groupings was reported.

### PsB and ctPsB model

In our previous work (9), the dynamics of the CML bulk transcriptome were modeled with three coupled variables: *BCR::ABL* expression, PBMCs, and the transcriptome state, defined as the position along the PCA-derived state-space. In this model, BCR::ABL acts as an upstream driver that perturbs the PBMC population, and the PBMC population and transcriptome form a mutual inhibitory feedback: cellular homeostasis suppresses transcriptome dysregulation, whereas increasing transcriptome dysregulation reduces the PBMC population. This is because changes in the transcriptome state are interpreted as destabilization of gene regulatory networks, consistent with a tristable attractor structure observed in the transcriptome state-space and compatible circuit motifs described by Dey and Barik (6). We applied the same modeling framework here to mathematically investigate how PsB transcriptome (U) dynamics created by cell type populations ($A_{i}$) driven by *BCR::ABL* expression (C) can combine with cell type weights ($w_{i}$) to form the PsB transcriptome as

$$\frac{dU}{dt}=\frac{k_{A1_{U}}}{1+{\left(\frac{A_{1}}{{A_{1}}_{U0}}\right)}^{n_{{A_{1}}_{U}}}}\cdot w_{1}+\;\frac{k_{A2_{U}}}{1+{\left(\frac{A_{2}}{{A_{2}}_{U0}}\right)}^{n_{{A_{2}}_{U}}}}\cdot w_{2}+\frac{k_{A3_{U}}}{1+{\left(\frac{A_{3}}{{A_{3}}_{U0}}\right)}^{n_{{A_{3}}_{U}}}}\cdot w_{3}+\;\frac{k_{A4_{U}}}{1+{\left(\frac{A_{4}}{{A_{4}}_{U0}}\right)}^{n_{{A_{4}}_{U}}}}\cdot w_{4}+\;\frac{k_{S_{UU}}\cdot {\left(\frac{U}{U_{U0}}\right)}^{n_{U_{U}}}}{1+{\left(\frac{U}{U_{U0}}\right)}^{n_{U_{U}}}}-\gamma_{U}\cdot U.$$

**Figure 4.**
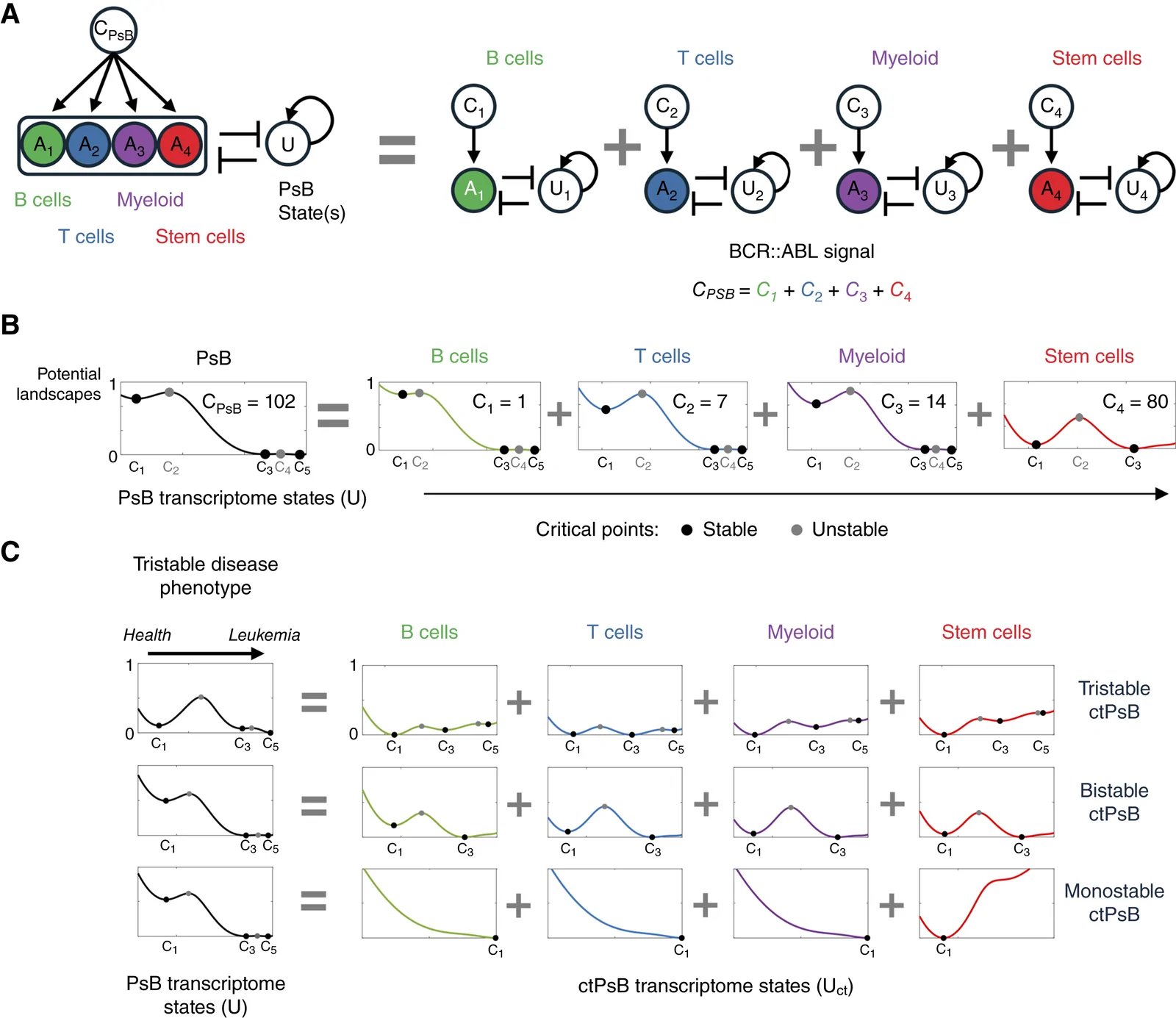
Math model relates cell type dynamics to the disease phenotype. **A,** Cell type PsB state-space dynamics were incorporated into a mechanistic model that related the total *BCR::ABL* CML oncogene signal computed from the PsB ($C_{PsB}$) to the PBMC population cell types ($A_{ct}$) and the PsB transcriptome state-space (U). The PsB state-space was reconstructed by adding each ctPsB model together as linear combination of each ctPsB model. The ctPsB models had cell type–specific parameters, including the *BCR::ABL* signal ($C_{ct}$) which was based on the observed *BCR::ABL* expression in each cell type. The total *BCR::ABL* signal in the PsB ($C_{PsB}$) was the sum of the signals from each cell type. **B, ***BCR::ABL* expression in each cell type and ctPsB state-space dynamics observed from the data informed the parameter used to construct a CP CML model. In this model, the cell type dynamics are combined to reconstruct the three-well potential that describes the PsB state-space phenotype dynamics. **C,** Mechanistic model demonstrates how different cell type dynamics can combine to produce tristable dynamics in the PsB phenotype. Even simple monostable cell types can produce a tristable phenotypic dynamics.

The overall PsB is obtained by setting $w_{1}=w_{2}=w_{3}=w_{4}=1$ (Fig. 4A), and ctPsB transcriptomes are produced by setting the value of $w_{i}$ to 0 or 1 as follows:

B cells ($A_{1}$): $w_{1}=1,\;w_{2}=w_{3}=w_{4}=0.$

T cells: ($A_{2}$): $w_{2}=1,\;w_{1}=w_{3}=w_{4}=0.$

Myeloid cells ($A_{3}$): $w_{3}=1,\;w_{1}=w_{2}=w_{4}=0.$

Stem cells ($A_{4})$: $w_{4}=1,\;w_{1}=w_{2}=w_{3}=0.$

Individual cell types evolve independently according to the following equations:

$$\frac{dA_{1}}{dt}=\frac{k_{C_{A1}}\cdot {\left(\frac{C}{C_{A10}}\right)}^{n_{C_{A1}}}}{1+{\left(\frac{C}{C_{A10}}\right)}^{n_{C_{A1}}}}+\frac{k_{U_{A1}}}{1+\;{\left(\frac{U}{U_{A10}}\right)}^{n_{U_{A1}}}}-\gamma_{A1}\cdot A_{1}$$

$$\frac{dA_{2}}{dt}=\frac{k_{C_{A2}}\cdot {\left(\frac{C}{C_{A20}}\right)}^{n_{C_{A2}}}}{1+{\left(\frac{C}{C_{A20}}\right)}^{n_{C_{A1}}}}+\frac{k_{U_{A2}}}{1+\;{\left(\frac{U}{U_{A20}}\right)}^{n_{U_{A2}}}}-\gamma_{A2}\cdot A_{2}$$

$$\frac{dA_{3}}{dt}=\frac{k_{C_{A3}}\cdot {\left(\frac{C}{C_{A30}}\right)}^{n_{C_{A3}}}}{1+{\left(\frac{C}{C_{A30}}\right)}^{n_{C_{A3}}}}+\frac{k_{U_{A3}}}{1+\;{\left(\frac{U}{U_{A30}}\right)}^{n_{U_{A3}}}}-\gamma_{A3}\cdot A_{3}$$

$$\frac{dA_{4}}{dt}=\frac{k_{C_{A4}}\cdot {\left(\frac{C}{C_{A40}}\right)}^{n_{C_{A4}}}}{1+{\left(\frac{C}{C_{A40}}\right)}^{n_{C_{A4}}}}+\frac{k_{U_{A4}}}{1+\;{\left(\frac{U}{U_{A40}}\right)}^{n_{U_{A4}}}}-\gamma_{A4}\cdot A_{4}$$

The equations are illustrated in Supplementary Fig. S5 with parameters and values given in Supplementary Table S4. In the equations, $k_{C_{Ai}}$ and $k_{U_{Ai}}$ represent the maximum contribution of cell population $A_{i}$ due to C, the bifurcation parameter that represents *BCR::ABL* expression level, and U, respectively. $C_{Ai0}$ represents the threshold value of C at the half-maximum of $A_{i}$, and $U_{Ai0}$ represents the threshold value of U at half-maximum of $A_{i}$. $n_{C_{Ai}}$ and $n_{U_{Ai}}$ represent the Hill coefficient on $A_{i}$ due to C and U, respectively. The Hill coefficient on U due to $A_{i}$ and itself is given by $n_{{A_{i}}_{U}}$ and $n_{U_{U}}$, respectively. ${A_{i}}_{U0}$ and $U_{U0}$ are the thresholds of $A_{i}$ and U, respectively, at the half-maximum value of U. $k_{Ai_{U}}$ is the maximum contribution to U due to the cell population $A_{i}$ and $k_{S_{UU}}$ is the maximum level of U due to its self-activation. The parameter $\gamma_{x}$ is the degradation rate constant of variable x.

The general form of the equations governing the dynamics between the number of cells of type i, *BCR::ABL* (C), and the transcriptome (U), are given by

$$\frac{dA_{i}}{dt}=g\left(C,U\right)-\gamma_{A_{i}}\cdot A_{i.}$$ (A)

$$\frac{dU}{dt}=h\left(A_{i},U\right)-\gamma_{U}\cdot U.$$ (B)

The steady state equation of $A_{i}$ is written as

$$A_{i}=\frac{g\left(C,U\right)}{\gamma_{A_{i}}}=g^{\prime }\left(C,U\right).$$ (C)

Substituting the steady state equation of $A_{i}$ in U using a transfer function, we obtain

$$\frac{dU}{dt}=h\left(g^{\prime }\left(C,U\right),U\right)-\gamma_{U}U.$$ (D)

Integrating the negative of Eq. 4 from 0 to U gives the effective potential function

$$V\left(C,U\right)=\;-\overset{U}{\underset{0}{\int }}\left[h\left(g^{\prime }\left(C,x\right),x\right)-\gamma_{U}x\right]dx.$$ (E)

Following the method introduced by Dey and Barik (13), using Eq. 5, we first generated bifurcation diagrams for the ctPsB and PsB models. Using these bifurcations with the heuristically chosen parameter values (Supplementary Table S4), we generated potential landscapes (Fig. 4B). Using different bifurcations and parameter values, we demonstrated how it is possible for PsB dynamics to be tristable when ctPsB landscapes are all tristable, bistable, or even monostable (Fig. 4C).

### Antagonistic teams of genes analysis

The top 90th percentile of eigengene magnitudes were selected from the CP CML state-space resulting in 2,951 genes. A gene-by-gene correlation matrix was made using the expression of these top eigengenes in all the leukemic mice samples. Clustering the gene-by-gene correlation matrix showed two groups of genes that are strongly correlated within the group and strongly anticorrelated with genes from the other group, which suggests mutually antagonistic teams (14). The direction of each eigengene was used to label each gene as pro- or anti-CML (Supplementary Fig. S7A). The same eigengenes were used for each cell type, and the SC-level gene expression for all leukemic samples was used to create a gene-by-gene correlation matrix for each cell type. Many of the genes had weak correlation, so the dendrogram was cut into three branches and the largest branch was removed. Heatmaps were produced to visualize the gene correlations from the remaining two branches to show different genes forming mutually antagonistic teams of genes for each cell type (Supplementary Fig. S7B). Each gene was labeled pro- or anti-CML based on the sign of the eigengene in the PsB state-space and in each corresponding ctPsB state-space (Supplementary Fig. S3D).

## Results

### CML state-transition is not detectable at the single-cell level

Our previous state-transition work constructed a CML state-space from time-series bulk RNA-seq (transcriptome) data (9). Using PCA by performing SVD on the time-series gene expression matrix, we showed that disease trajectories were encoded in the CML state-space corresponding to the first PC (PC1; Supplementary Fig. S1A; refs. 9, 15). The state-space comprised a continuum of transcriptomic states that spanned from health to disease.

To test how a state-transition model could also be derived and whether it could be more informative using transcriptomes of individual cells, we conducted a time-series scRNA-seq analysis on PBMC samples collected over time from a *BCR::ABL* inducible mouse model of CP CML (9). Mouse peripheral blood was sampled before induction of *BCR::ABL* by tet withdrawal at week 0 (T_0_) and then weekly for 10 weeks after induction (11 samples per mouse; 29 total samples; Fig. 1A). Because the mice developed CML at different rates, we scaled the time points to represent a progression from the first time (T_0_) to the last collection time point (T_f_), which was either before a moribund mouse was culled or end of the study (week 10), whichever occurred first. We used the scaled time to identify whether a candidate state-space ordered the mouse time point samples from the healthy time point (T_0_) to the leukemic time point (T_f_).

To construct a single-cell leukemia (or CML) state-space, we applied SVD to the SC gene expression matrix and visualized scaled time in each resulting PC (Fig. 1A). One of the *BCR::ABL*-induced mice did not develop leukemia over time and therefore did not have a leukemic time point (T_f_). We observed that the first two PCs grouped cells by cell type rather than by disease status, and, in fact, none of the first six PCs, UMAP, or diffusion map analysis could separate cells by disease state (i.e., health vs. leukemia; Supplementary Fig. S1B and S1C). We therefore concluded that the difference in cell types, rather than in disease status, was the primary source of variance in the SC transcriptomes. Thus, we examined whether leukemia state-space could be identified by analyzing each cell type independently. To this end, we categorized the PBMCs into four main cell types: B, T (including NK cells), myeloid, and stem cells based on cell type annotation analysis (Fig. 1A; Supplementary Table S1). When SVD was performed on the SC gene expression matrix of each of the four cell types individually, no PC for any of the cell types produced a state-space that separated health from leukemia; in fact, we did not observe any unique leukemic transcriptional states in the T_f_ cells (Fig. 1B; Supplementary Fig. S1D). Other methods for scRNA-seq analysis (10, 11) were also unable to identify a state-transition from heath to leukemia at the SC-level (Supplementary Fig. S1E and S1F). Of note, despite not observing a unique leukemic state-space at the SC-level, we observed DEGs in each cell type when gene expression at T_0_ was compared with that at T_f_, indicating that individual gene expression does change during leukemia progression but not sufficiently to overcome the cell type signature or to identify the system phenotype transition in the SC transcriptomes (Supplementary Fig. S1H).

Taken together, these results suggest that, from T_0_ to T_f_, the cells occupied a continuum of transcriptional states that always overlapped healthy and leukemia states (Fig. 1B; Supplementary Fig. S1B–S1F). We describe this continuum of transcriptional states in each lower dimensional representation of the data as a superposition of cell states.

### CML disease evolution is observable in the PsB

As no leukemic state emerged from the SC-level transcriptome analysis, we then asked whether the individual cells when aggregated can reveal the disease phenotype. To examine whether disease phenotype dynamics encoded by the cell microstates could be identified when we considered these microstates in aggregate (i.e., macrostates), we constructed PsB transcriptome samples by combining gene counts from all individual cells. After performing SVD on the PsB gene expression matrix, we observed a trajectory of the PsB transcriptomes from healthy state (T_0_) to leukemic state (T_f_) in PC1 (Supplementary Fig. S2A and S2B). This trajectory was also the only PC that correlated with BCR::ABL expression levels (Supplementary Fig. S2B; Supplementary Table S2) and therefore identifiable as the PsB CML state-space, which had dynamics similar to the bulk CML state-space and could be described by five critical points ($c_{1-5}$; Fig. 1C). We then aligned the PsB CML state-space and a bulk CML state-space that we previously reported (9), and plotted them together over time to compare how their respective disease dynamics mapped in the state-space (Supplementary Fig. S2C). Like in the bulk state-space (9), the disease dynamics in the PsB state-space was described by a leukemogenic potential with three steady states (or “wells”) and therefore could be produced by the same mathematical model that was used to derive the bulk state-space (Fig. 1D; ref. 9). The three wells of the leukemogenic potential were used to define three disease states of leukemia (stable critical points), and we labeled them as follows: early state (Es) at $c_{1}$, transition state (Ts) at $c_{3}$, and late state (Ls) at $c_{5}$. The three-well potential also had two unstable critical points which served as the boundaries between the steady states and that we labeled as follows: early transition state (T-Es) at $c_{2}$ and late transition state (T-Ls) at $c_{4}$.

### CML disease evolution is not revealed by single-cell data

Because CML progression was readily detected in the PsB data, we next asked whether an analogous disease trajectory is encoded in the scRNA-seq data. We quantified the extent to which time, as it is associated with CML progression in our time-series data, and *BCR::ABL* expression, as it is a direct proxy for leukemic burden, explain variation along the leading PCs of the SC-level data. Specifically, for each PC, we computed both the marginal *R*^2^ (PC modeled as a function of each variable alone; Supplementary Fig. S3A) and the partial *R*^2^ which computes the additional variance explained by each variable after controlling for other sources of variance including mouse identity and cell type (Fig. 1E). In the SC data, time and *BCR::ABL* showed weak associations with PCs (*R*^2^≤ 0.1), whereas cell type was strongly associated with PC1 and PC2 [Fig. 1E (left)]. In contrast, in the PsB data, time was strongly associated with the leading disease axis [PC1; *R*^2^ = 0.80; Fig. 1E (right)], consistent with a longitudinal disease trajectory at the macrostate level.

To ensure that cell type composition was not masking within-lineage disease trajectories, we repeated the same PC association analysis within each of the four main cell types, again observing weak associations with time and *BCR::ABL* across PCs (Supplementary Fig. S3B). Finally, we summarized the fraction of variance explained by each variable in each data level and confirmed that time is a significant source of variation in PsB data but a small source in SC-level data [Fig. 1F; Supplementary Fig. S3A]. Together, these results indicate that unlike the PsB macrostate representation, SC-level transcriptomes do not contain a clear signature of CML progression in this dataset, and therefore, the CML disease phenotype cannot be consistently detected at the single-cell level.

### Each cell type undergoes CML state transitions

Based on the observation that the CML state transition was encoded by the total PsB data, we then tested whether we could identify a state-space that encoded the transition from health to leukemia also using individual cell type PsB transcriptomes. To this end, we created PsB transcriptome samples for each of the four PBMC cell types (ctPsB) and performed SVD separately on each ctPsB gene expression matrix (Supplementary Fig. S3C). Using the scaled time ordering of the samples and *BCR::ABL* expression for each of the four cell types, we were able to identify the CML state-space in either the first or second PC for each of the four cell types (Fig. 2A). Like the PsB state-space, the B, T, and myeloid ctPsB all had tristable potentials (Supplementary Fig. S3D). In contrast, likely due to the smaller number of cells in each sample, the trajectories for the stem cells were noisier and seemed to exhibit bistable dynamics (Supplementary Fig. S3D). This result demonstrated that individual cells indeed contained information about disease state-transition that can be extracted only when the SC-level microstates are considered in aggregate using PsB transcriptomes.

To explore how each ctPsB contributed to the total PsB state-space, we compared the trajectory of the total PsB transcriptomes with those of individual ctPsB transcriptomes mapped into the total PsB state-space (Supplementary Fig. S3E). We noted that each projected ctPsB trajectory started and ended at different locations of the state-space. To summarize the ctPsB projections, we defined the span of their trajectories to be the minimum and maximum observed state-space coordinates for each cell type (Supplementary Fig. S3F). We noted that the span of each cell type only occupied a subset of the PsB trajectories and that each span had different lengths. Although none of individual ctPsB trajectories fully spanned the total PsB state space, when considered together, they completely overlapped the total PsB state-space, suggesting that the latter is a linear combination of the former and that each cell type contributed differently to the disease dynamics.

### State transition reveals cell type–specific contributions to CML development

To understand the contribution of individual cell types to disease dynamics, we then plotted the proportions of each cell type over the scaled time for each leukemic mouse to visualize the changes that occurred during CML development (Fig. 2B). To this end, we grouped the samples into the three disease states (Es, Ts, and Ls) using their state-space coordinates and compared the cell type proportions of the individual cell types at each disease state with those observed at the healthy (T_0_) state (Supplementary Table S3; Fig. 2B). The stem cells were the first to show significant population changes that started at Ts (increased; *P* < 0.001) and continued to increase to Ls (*P* = 0.001). The myeloid and B cells did not change until Ls with the myeloid cell population increasing (*P* = 0.03) and B-cell population decreasing (*P* = 0.03). The T/NK-cell population did not show significant changes.

Next, we performed a computational simulation that leveraged both the time-series samples and the PsB state-space to quantify the contribution of each cell type to CML development. To simulate the effect of not having one of the cell types evolve over disease development, we computationally subtracted the contribution of each cell type by fixing both the cell counts and gene expression of each of the cell types individually to the values observed at the healthy state (T_0_; Fig. 2C). We projected all time points of the “fixed cell type” data into the space to construct simulated trajectories of the “fixed cell type” PsB transcriptomes. We then compared each “fixed cell type” simulated trajectory to the experimentally derived (observed) PsB transcriptome trajectories (Fig. 2D). To quantify the contribution of each cell type to the state-transition from health to leukemia, we calculated the difference between the simulated and observed trajectories at each time point [Fig. 2D (middle)]. We postulated that if a fixed cell type did not shorten the state-space trajectories compared with the observed PsB trajectories, then that fixed cell type likely did not contribute significantly to the PsB state-transition from health to leukemia. Conversely, we interpreted a larger distance between the observed and simulated trajectories as lost information due to the lost contribution of the fixed cell type.

To quantify information loss, we calculated the Kullback–Leibler divergence between the observed and the “fixed cell type” simulated PsB trajectories for each cell type [Fig. 2D (right)]. We observed that fixing the myeloid cells was associated with the largest information loss, suggesting that this cell population contributed the most to CML state-transition. This result was also supported by the observation that myeloid cells also had the largest span between the projected ctPsB and PsB trajectories in the state-space (Supplementary Fig. S3F) and therefore the largest contribution to CML state-transition which is likely due to the combination of the progressively increasing cell number (Fig. 2B) and the gene expression changes (Supplementary Fig. S1H) over time. Although the myeloid cells contributed the most information, we noted that all cell types contributed information to the disease phenotype, which further supports our macroscopic view of disease.

### An independent CML model confirms the state-space is encoded in PsB data

To confirm our findings, we conducted a second time-series scRNA-seq experiment using a different mouse model that recapitulates BC CML development (16). Although the CP CML mouse was a *BCR::ABL*-driven inducible model, the BC mouse, in addition to inducible *BCR::ABL* expression, had a germline *Mir142* knockout as a “second leukemogenic hit” that triggered transformation. Although genotypic differences between these two models prevented direct comparison of the transcriptional and disease states, we used the same analytic approach to identify a SC-level state-space for the BC model.

Like the CP model, we were unable to identify state-transition encoded in the SC-level transcriptomes, but we could identify a leukemic state-space in PC1 when SC information was aggregated into the PsB transcriptomes (Supplementary Fig. S4A–S4C). Based on the BC CML PsB state-space trajectories, we observed that BC CML exhibited bistable phenotypic dynamics that were described by a two-well potential labeled as early and late disease states (Supplementary Fig. S4D). When we constructed ctPsB for the BC CML samples, we observed that each cell type encoded state-transition and spanned different portions of the PsB state-space (Supplementary Fig. S4E–S4G). Although BC CML has different dynamics when compared with CP CML, the BC CML experiment validated our main findings that the leukemic state-transition is not encoded at the SC microstate level but is observed only when PsB macrostates are considered.

Finally, we applied the fixed cell type simulation strategy to the BC CML samples to quantify the contribution of each cell type to BC CML PsB state-space [Fig. 2E (bottom)] using Kullback–Leibler divergence to assess the difference in information between the simulated versus PsB trajectories. In the BC CML model, we observed that B cells and myeloid were the cell types that contributed the most information to state transition [Fig. 2E (right)].

### Validation of the macroscopic state-transition in human CML

Our experimental setup had two potential confounding factors: (i) in our mouse model, *BCR::ABL* is induced in every cell type which could homogenize the cell state changes and (ii) profiling may underrepresent the transformed progenitor/stem compartments that define disease. To address both in the case of natural progression, we analyzed CD34^+^ selected BM scRNA-seq from patients with CML in CP at diagnosis and age-matched healthy controls (*n* = 4 CML, *n* = 3 healthy). As in the mouse experiments, PCA and other dimensionality reduction methods at the single-cell level did not reveal a disease-specific transcriptional state. When all cells were analyzed together, PCA and UMAP both separated cells by lineage rather than disease state (Fig. 3A; Supplementary Fig. S5A and S5B). The same was true when we analyzed each cell type independently which showed that in the top five PCs, the healthy and CML cells occupied overlapping, continuous transcriptional states (Fig. 3B; Supplementary Fig. S5C). Thus, we concluded that disease status was not encoded as discrete single-cell states in the human BM data. In contrast, when we aggregated cell counts into PsB samples, the disease macrostate emerged as a unique transcriptional state. PC1 of the PsB PCA identified a state-space that could cleanly separate CML from healthy samples (Fig. 3C). Together, these results reproduce what we observed in the mouse models: the cell level data constitute microstates of the disease phenotype macrostate.

Because these data are from different individuals, we adapted our fixed cell type simulation to quantify contributions without matched time-series samples. We used all healthy–CML combinations, and for each pairing and each cell type, we “fixed” that cell type by replacing its CML cells with the paired healthy cells (Supplementary Fig. S5D). We then recomputed the PsB state-space for the fixed simulations on each cell type [Fig. 3D (left)] and measured the shortfall in the state-space distance relative to each healthy–CML difference [Fig. 3D (middle); Supplementary Fig. S5D]. The information lost from all fixed simulation combinations was calculated using the Kullback–Leibler divergence to show that common myeloid progenitors and pro-B cells contributed the largest share of information to the human CML, whereas the pooled “other” category contributed negligible information (Fig. 3D). Similar to the mouse results, the fixed simulation on human BM data confirms that multiple cell types contribute information to the CML macrostate.

### Cell type contributions are modeled as linear combinations of their transcriptomic state

We showed that each of the PBMC cell types contribute in different proportions to the CML state-transition. To model the dynamics of state-transition mathematically, we postulated that the total *BCR::ABL* signal from the PsB ($C_{PsB}$) acts on each cell type as it is constitutively expressed in all cells in the mouse model and affects their ctPsB transcriptome (Fig. 4A) as

$$\frac{dA_{ct}}{dt}=G_{A_{ct}}\left(C_{ct},U\right)-k_{A_{ct}}A_{ct,\;}\frac{dU}{dt}=\;\underset{ct}{\sum }G_{U}\left(A_{ct}\right)+F_{U}\left(U\right)-k_{U}U.$$

Here, $A_{ct}$ represents the number of each of the four cell type populations, which are assumed to contribute to the PsB independently, and U is the PsB transcriptomic state (13). The functions $G_{A_{ct}}\left(C_{ct},U\right)$, $G_{U}\left(A_{ct},U\right)$, and $F_{U}\left(U\right)$ are composed of Hill functions of the form $G_{A_{ct}}\left(C_{ct},U\right)=H^{+}\left(C_{ct}\right)+H^{-}\left(U\right)$, $G_{U}\left(A_{ct}\right)=\;w_{ct}H^{-}\left(A_{ct}\right)$ and $F_{U}\left(U\right)=H^{+}\left(U\right)$, in which $H^{+}$ represents a Hill function for activation and $H^{-}$ represents a Hill function for repression (see “Materials and Methods”). $w_{ct}$ is a cell type weight factor that determines the contribution of each cell type to the PsB. The effective potential of the system (13) is the integral of the leukemic “force” which depends on the *BCR::ABL *signal, so that

$$V\left(U,C_{PsB}\right)=-\int_{0}^{U}\left[\underset{ct}{\sum }G_{U}\left(G_{A_{ct}}\left(C_{ct},x\right)\right)+F_{U}\left(x\right)-k_{U}x\right]dx.$$

To apply this model to our CP CML scRNA-seq data, we used both the dynamics observed in each ctPsB state-space (Fig. 2A) and the BCR::ABL expression in each cell type (Supplementary Fig. S3G) to determine the number and location of the steady states for each cell type (Supplementary Fig. S3D). From the critical points, we constructed the potentials that define state-transition dynamics for each cell type (Fig. 4B). Based on the data, we modeled the B, T, and myeloid cells as having tristable dynamics and stem cells as bistable. Individual cell type models were constructed by heuristically chosen parameters that controlled the cell type dynamics so that each cell type model matched the observed ctPsB state-space and projected dynamics (Fig. 4B; Supplementary Fig. S6A; Supplementary Table S4).

The resulting model recapitulated a tristable CML potential constructed from the cell type transcriptional dynamics, suggesting that the disease phenotype dynamics could be produced from a linear combination of the ctPsB. Moreover, we noted that the tristable potential observed in the system-level PsB state-space could be produced using combinations of different cell type dynamics, including cell type potentials that were either all monostable, bistable, or tristable (Fig. 4C). Here, the theoretical model reinforced our understanding of the information hierarchy embedded in disease state transitions. Although certain cell types exhibited tristable and bistable states, cell type dynamics could also manifest as simpler monostable states, in which cells transition from their original stable state to occupy a single new state. These dynamics can again be aggregated using the model to produce system-level phenotype dynamics, which describes the disease trajectories of individuals.

## Discussion

In this study, we studied the evolution of CML from the point of view of an epigenetic state-transition of the peripheral blood transcriptome. Although it is widely recognized that cells undergo state transitions during normal and pathologic differentiation (17–24), we studied this process with longitudinal sequencing data from a mouse model of CML with the goal of understanding the relationship between single-cell state transitions and phenotype transition from health to disease. scRNA-seq data present significant analytic challenges, including limited read depth, dropout events, and complexities in interpreting longitudinal dynamics (bioRxiv 2022.06.03.494765; refs. 25–30). For these reasons, we guided our experimental and analytic approach with a theoretical and mathematical framework (9, 15, 31). We first showed that CML transcriptional state-transition was not encoded in the cell level data, in which cells were found to exist in a continuum of transcriptional microstates in a superposition of health to disease states that were not correlated with *BCR::ABL* or time. Instead, the phenotype state-transition became apparent only after information derived from single-cell microstates was integrated into macrostates by constructing PsB transcriptomes.

To explore state transitions in different cell populations, we performed PsB for each cell type (ctPsB) and showed that each cell type had both a unique state-space and distinct CML state-transition dynamics. We developed a computational simulation to quantify each cell type’s contribution to CML by fixing each cell type population at its healthy state for all subsequent time point samples and determined information loss by comparing each fixed cell type simulation to the experimentally observed PsB trajectories. In our CP mouse model, we identified myeloid cells as the cell type that contribute the largest amount of information to the system dynamics. Although this is not surprising given that we used a CML mouse model, it is important to underscore that this conclusion was derived experimentally from an unbiased data analysis, thereby validating the usefulness of this approach to CML and its potential applicability to other types of leukemia and cancer to predict disease evolution or treatment response (9). To this end, these state-transition results were validated in a second time-series experiment on a BC CML model and found to be consistent with analysis of CD34^+^ selected cells from human BM. Despite differences in genotype and CML disease model, both experiments demonstrated that the SC level data encoded a microstate of the PsB phenotype dynamics. Finally, we studied a phenomenologic mathematical model in which the PsB transcriptome dynamics could be recapitulated as a linear combination of the ctPsB dynamics and the cell type–dependent effects such as *BCR::ABL* expression. The model provided a theoretical framework to understand how cell types with different transcriptional dynamics can produce complex system-level phenotype dynamics.

Taken together, we illustrate here how disease-relevant information is contained from data collected at different biological scales. We used the state-transition model to discover a relationship between SC- and PsB/system-level data. We described this relationship using the thermodynamic concepts of microstates and macrostates as previously hypothesized (32–34) but not yet experimentally demonstrated through longitudinal single-cell analysis (35). At the SC-level, cells exist in a continuum of microstates that include both healthy and disease transcriptional cell states. Although we were unable to recover disease state transition from SC microstates, the system phenotype (i.e., disease transition) can be recovered at the macrostate level when SC microstates are considered in aggregate (i.e., PsB). Thus, the macrostate may be considered as a continuous variable that describes phenotype evolution within a state-space that ranges from healthy to disease. For both CP and BC CML models, we showed that the macrostate could be identified using either the PsB data from all PBMCs or using the ctPsB data from any one of the cell types that comprise the PBMCs. Given a PsB transcriptome from any of these sources, we also discovered that the disease macrostate emerged from aggregated SC microstates, enabling prediction of phenotype evolution from the epigenomic state (36, 37). This microstate/macrostate model is analogous to the thermodynamic situation in which one can measure the energy of a single particle (microstate), but that measurement does not provide any information about the temperature of the system as a whole (macrostate). Like energy and temperature in thermodynamics, we have shown that the CML disease phenotype is encoded into a transcriptome macrostate that is defined by the ensemble of cell microstates.

As the disease macrostate was identified as a one-dimensional projection of the full transcriptome, it was constructed from a linear combination of all 29,513 expressed genes. In contrast to mathematical models that use cell proliferation and differentiation to model CML and other hematologic malignancies (38–43), our approach, which aggregates scRNA-seq data, implicitly integrates both cell population and gene expression information. Dynamic system-based approaches have been widely used to explore cell dynamics in single-cell expression data (10, 44–47). Although our approach is related due to conceptualizing state transitions as a landscape, there is a fundamental difference between biological dynamics which can be generally categorized into either cell versus system phenotype dynamics.

One difference between these two types of dynamics is the time scale on which they occur. Cell phenotype (i.e., differentiation or development) involves shorter time scales in which dynamics occur on the order of a single cell’s division and is the focus of existing single-cell approaches. System phenotypes (i.e., disease evolution) instead have dynamics that occur on the order of many cell generations. Although cell phenotype dynamics often are identifiable or defined by the state of an individual cell, we propose that system phenotypes cannot be defined by a single cell (microstate) and instead represent biological macrostates.

Limitations of our modeling approach include the assumption that the system state-transition follows a gradient flow along a potential landscape, which does not permit oscillatory dynamics that may be observed in rare cases of CML (48–54). Additionally, our modeling framework does not explicitly include molecular mechanisms for how biological macrostates are produced; however, recent work has shown that disease dynamics may be derived from models of proliferation and differentiation in a single-cell state-space (43). It is also possible that phenotypic macrostates may be produced by steady-state switching in gene regulatory networks as shown in recent literature (14, 55, 56). A hallmark of transcriptomic data that produces strongly defined states is the existence of both low dimensional and antagonistic teams of genes which are defined by anticorrelated expression among genes in the network (56). In our PsB CP CML data, we observed that the top eigengenes had a gene expression correlation structure that was similar to antagonistic teams (Supplementary Fig. S7A). When we conducted the same analysis on the SC-level data for each of the cell types, we observed different genes forming antagonistic teams of genes in each cell type (Supplementary Fig. S7B; Supplementary Table S5). This may indicate that gene networks, and not the states of individual cells, are the mechanism that produces the macroscopic states we observed in the CML state-space.

Of note, we demonstrated that different PBMC cell types contributed varying amounts of information to the identification of CML development dynamics (Fig. 2D and E). This enabled us to develop a phenomenologic model that explains how the *BCR::ABL* oncogene, although necessary for CML development, differentially affects each cell type’s transcriptome and how these PsB cell types collectively reproduce the leukemia dynamics observed in bulk RNA data. Importantly, these observations applied to two different mouse models, one that recapitulated CP and the other BC. We observed that all the cell types had nonlinear dynamics similar to the observed disease dynamics in the PBMCs. However, one intriguing result is that even monostable cell type dynamics when combined could produce tristable system-level dynamics as those observed from the PsB and bulk transcriptome analysis. This modeling result mathematically demonstrates how simple, linear dynamics within individual cell types can generate complex, nonlinear phenotype dynamics, explaining how biological systems can exhibit emergent properties in which the whole exceeds the sum of its parts. Put another way, investigating the dynamics of a single-cell type does not necessarily inform you about the system-level disease dynamics. Additional future applications of our model could use *in silico* perturbations of the cell type weights to identify rare cell populations that have large contributions to disease progression using an approach similar to what Ramirez and colleagues (55) applied to gene networks. The cell types identified by the perturbation analysis could then experimentally be knocked down to confirm their role in disease and as new therapeutic candidates.

In conclusion, our findings establish a novel conceptual framework for understanding how cellular information generate phenotypic states that define disease state transitions, with wide ranging potential clinical applications in predicting disease development, transformation, treatment response, and ultimately patient survival. By quantifying how single-cell transcriptional microstates comprise disease-defining macrostates, our framework suggests a path toward predictive modeling that could inform clinical management of CML by identifying patients at the highest risk of progression or optimizing therapeutic strategies based on cell type state transitions. Future application of these models in patient care may enable more accurate predictions of disease progression and treatment outcomes, facilitating timely therapeutic intervention.

## Supplementary Material

Figure S1 Single cell analysis to identify CML state.

Figure S2 Construction of the CML state-space in PsB data.

Figure S3 CML dynamics in the sc, ctPsB, and PsB levels of the transcriptome.

Figure S4 Analysis of the BC CML model cohort.

Figure S5 Identifying a CML state-space in human CD34+ BM scRNA-seq.

Figure S6 Model of CML dynamics from cell type transcriptomes.

Figure S7 Characteristics of antagonistic teams of genes in PsB and sc-level data.

Table S1 Cell type counts and categories.

Table S2 PsB state-space loading values and correlation with BCR::ABL.

Table S3 Cell type proportion testing table.

Table S4 Parameter values for the model.

Table S5 Genes that produce anti-correlated teams.

## Data Availability

All scRNA-seq raw sequence reads and the Seurat object are available via the GEO for the mouse CP and BC CML (GSE296507) and dbGaP for the human (phs004729.v1.p1) experiments. All other raw data are available upon request to the corresponding authors.

## Code Availability

Code to perform all analyses, modeling, and to generate all figures are available at https://github.com/cohmathonc/CML.BC.scRNA-manuscript.
