# Supplementary material for: Longitudinal Single-Cell RNA Sequencing Reveals Evolution of Microstates and Macrostates in Chronic Myeloid Leukemia
Source: Cancer Res. 2026 Jun 22;86(21):5564–78. doi: 10.1158/0008-5472.CAN-25-4371 (PMC13679914; doi:10.1158/0008-5472.CAN-25-4371)
Supplement: Supplementary file 3 [file can-25-4371_figure_s3_suppsf3.pdf]

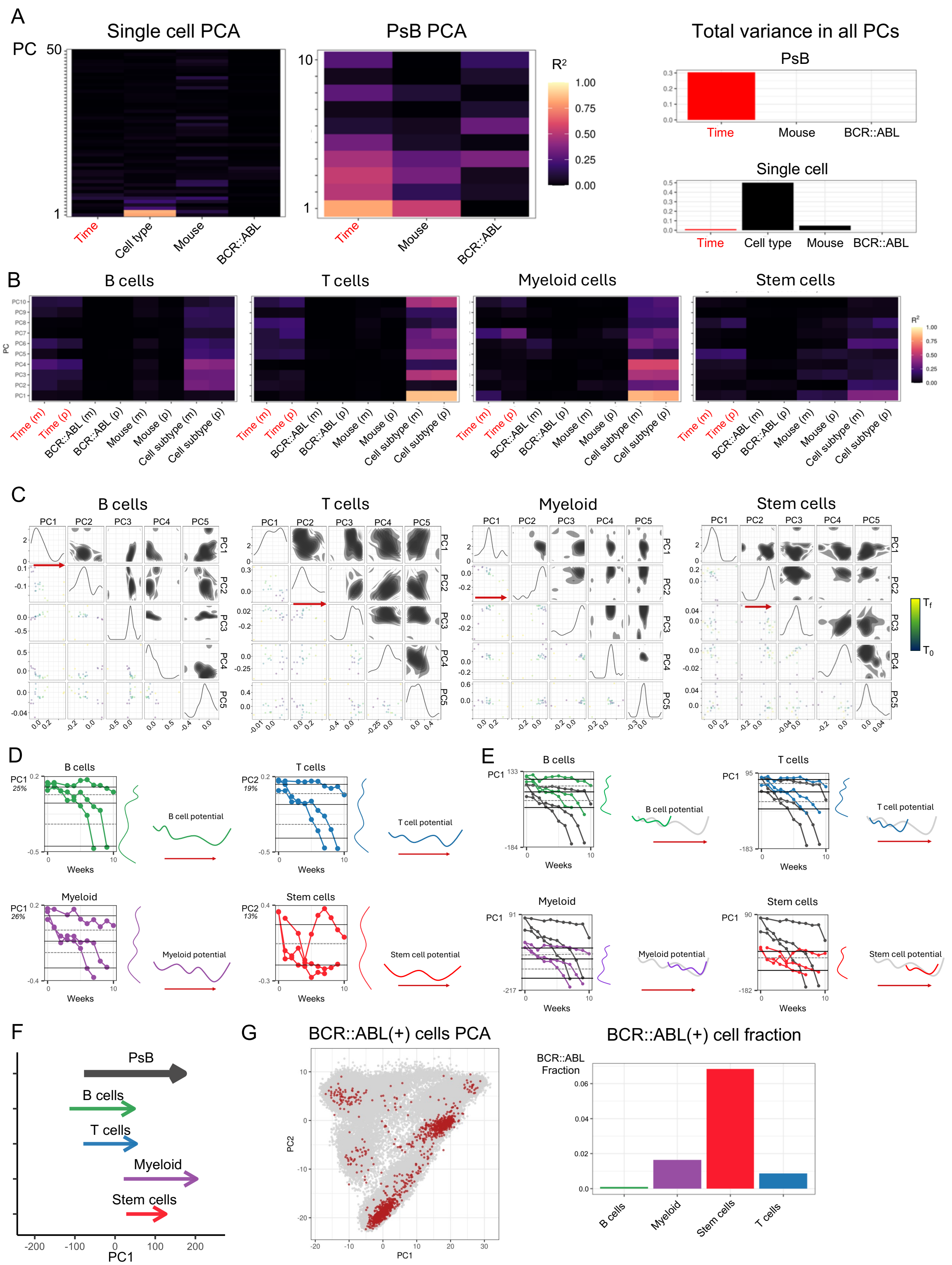

Figure S3: CML dynamics in the sc, ctPsB, and PsB levels of the transcriptome

A) Marginal regression was used to show the association between covariates and the SC and PsB level principal component (PC) structure. R^2 values are plotted in a heatmap for each data

for each PC (*left*). Time is highlighted as it was used to represent CML evolution. Variance attributed to each covariate was summarized by weighting the marginal R^2 by each PC's variance fraction and summing across all PCs (*right*). **B)** Heatmaps for both marginal (m) and partial (p) regression between each covariate and PCs of each cell type were also produced for each cell type. Cell subtypes represent the more granular cell labels within each cell type. **C**) SVD was performed on each cell type pseudobulk (ctPsB) gene expression matrix; the first five PCs were plotted using the scaled time colors, and the PC that encoded the ctPsB state-space was indicated (*red arrows*). **D**) For each ctPsB state-space, the trajectories were used to estimate the state-space dynamics for each cell type (percent of total variance explained by each PC shown under the y-axis label). The stable critical points (*black lines*) were drawn based on how many stable states were observed, and the unstable critical points (*grey dashed lines*) were drawn based on where the trajectories showed the highest velocity. Using these assumed critical points, an estimated potential was constructed to illustrate the dynamics of each cell type during leukemia progression (*red arrow*). The number of stable critical points in each cell type here were used to determine the number of steady-states in each cell type's bifurcation diagram in the cell type model (Fig. 4B). **E**) The ctPsB data for each cell type (*colored trajectories*) were projected into the PsB state-space (PsB trajectories in shown in black) by multiplying the ctPsB gene expression by the right singular values from the PsB SVD result (Fig. S2A). For each ctPsB projection, the stable critical points (*black lines*) were drawn based on how many stable states were observed, and the unstable critical points (*grey dashed lines*) were drawn based on where the trajectories showed the highest velocity. Using these critical points, an estimated potential was constructed to illustrate the dynamics of each cell type during leukemia progression (*red arrow*). The ctPsB potentials were shown with respect to the PsB potential (*grey*) constructed using the same critical point estimation approach. **F**) The extent and location of each ctPsB projected trajectories and the PsB trajectories were each summarized using the minimum and maximum PsB state-space coordinate. **G**) Cells with BCR::ABL expression [BCR::ABL(+)] were indicated in the PCA representation of all cells from all time points (*left*). The bar plot (*right*) shows the fraction of BCR::ABL(+) cells in each cell type.
