# Supplementary material for: Longitudinal Single-Cell RNA Sequencing Reveals Evolution of Microstates and Macrostates in Chronic Myeloid Leukemia
Source: Cancer Res. 2026 Jun 22;86(21):5564–78. doi: 10.1158/0008-5472.CAN-25-4371 (PMC13679914; doi:10.1158/0008-5472.CAN-25-4371)
Supplement: Supplementary file 4 [file can-25-4371_figure_s4_suppsf4.pdf]

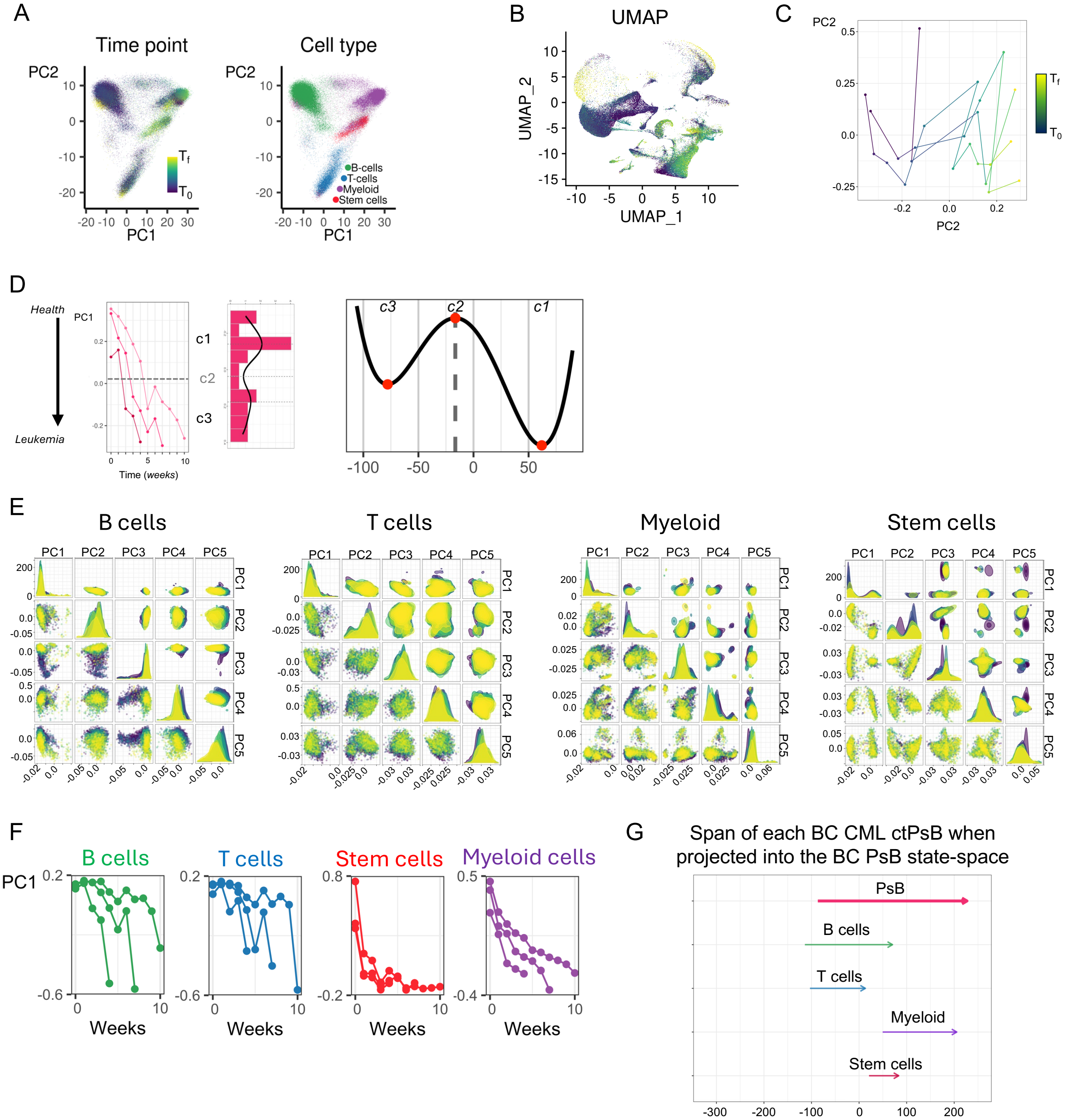

Figure S4: Analysis of the BC CML model cohort

A) Using all cells and all time point of the BC CML mouse experiment, PCA was performed on the mean-centered expression of all genes. The first two PCs were plotted for each cell, and the cells were colored by the sample's scaled time points (*left*) and cell type (*right*). **B**) UMAP was also performed on all cells, and the cells were colored by their samples scaled time. **C**) SVD was performed on the BC CML PsB gene expression matrix, and the mouse trajectories in the first two PCs were shown and colored by the scaled time. PC1 encoded the BC CML PsB state-space. **D**) The trajectories of each BC CML mouse are shown in the PsB state-space (*left*). The sample density in the PsB state-space was fit using kernel density estimate to determine the number of steady states and the location of the critical points (*middle*). The critical points were used to construct a potential function that describes the PsB BC CML dynamics. **E**) SVD was

performed on the SC-level gene expression separately for each cell type, and the sample's scaled time was shown used to color each cell in the first five PCs of each cell type. **F**) SVD was performed on the ctPsB data for each cell type and the mouse ctPsB trajectories were shown for PC1 which was identified as the ctPsB state-space for each cell type. **G**) The ctPsB gene expression data were projected into the PsB state-space for each cell type. The extent and location of the ctPsB trajectories and the PsB trajectories were summarized by taking the minimum and maximum PsB state-space coordinate for each set of trajectories.
