# Supplementary material for: Longitudinal Single-Cell RNA Sequencing Reveals Evolution of Microstates and Macrostates in Chronic Myeloid Leukemia
Source: Cancer Res. 2026 Jun 22;86(21):5564–78. doi: 10.1158/0008-5472.CAN-25-4371 (PMC13679914; doi:10.1158/0008-5472.CAN-25-4371)
Supplement: Supplementary file 5 [file can-25-4371_figure_s5_suppsf5.pdf]

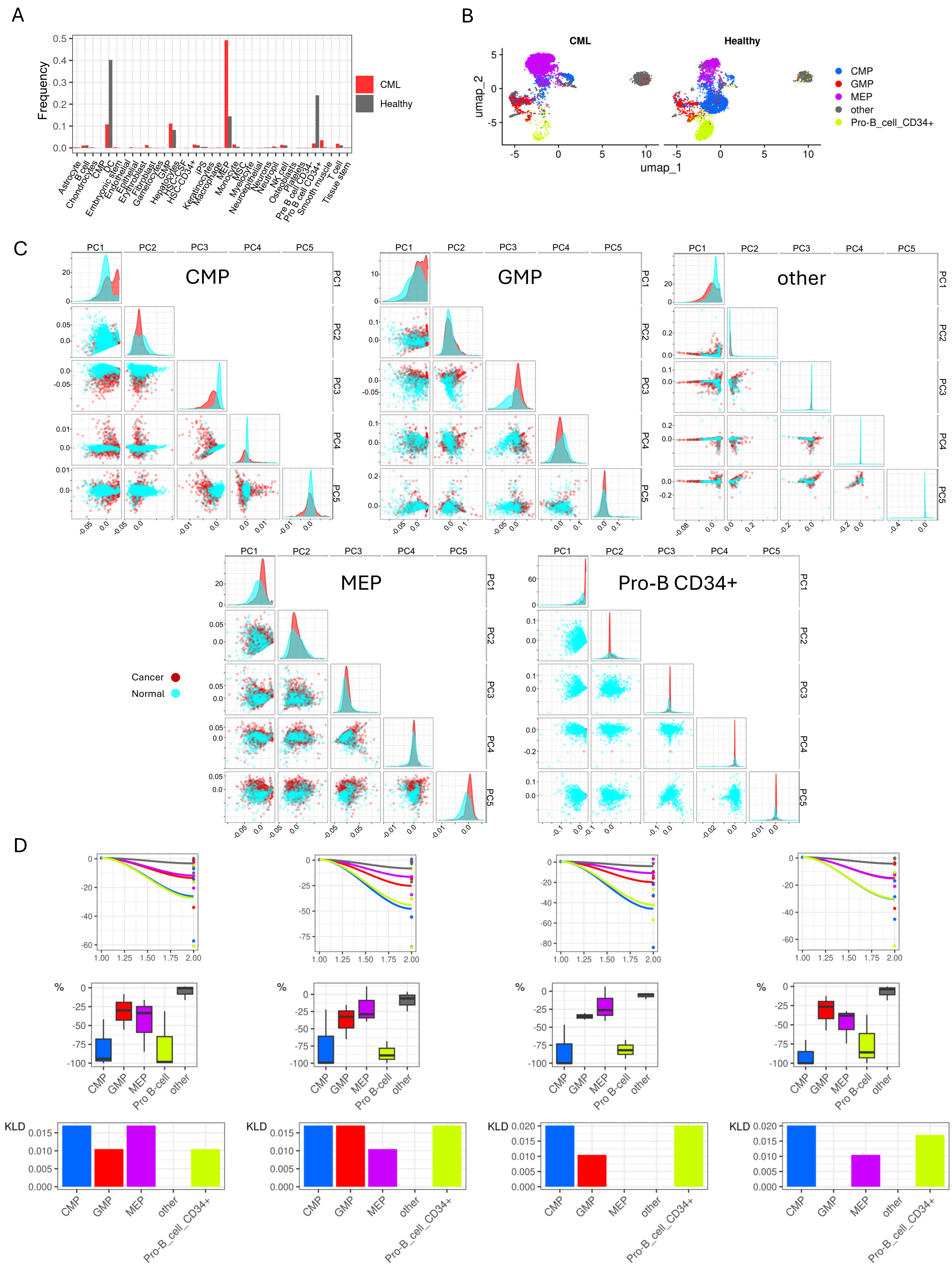

Figure S5: Identifying a CML state-space in human CD34+ BM scRNA-seq

A) From the human CD34+ BM scRNA-seq data, cell types were labeled and summarized as the fraction of total cells in each disease group. For the purposes of analysis, all cell types with less 5% in both healthy and CML disease groups were categorized as “other”. **B**) UMAP was performed on all cells, and the cells were colored by cell type. **C**) SVD was performed separately on each cell type, and the top five PCs were used to try to identify transcriptional cell states unique to the CML samples. **D**) To quantify how each cell type contributed to CML development, the unpaired samples were treated as healthy-CML pairs by matching each of the three healthy samples to one of the CML samples to create four different healthy-CML pair combinations. All possible combinations of sample pairings were used in the fixed simulations, and for each pairing and each cell type, the CML sample’s cells were replaced with the paired healthy sample’s cells to simulate what would happen if each cell type was fixed in a healthy state. The plots here quantify how fixing each cell type moved the location of the CML sample in the state-space toward the healthy state (top row) and the percent of the total distance observed for each healthy-CML pair when the cell types were not fixed at the healthy state (middle row). The information lost by fixing each cell type was computed using Kullbach-Leibler divergence (KLD; bottom row).
