# Supplementary material for: Longitudinal Single-Cell RNA Sequencing Reveals Evolution of Micro- and Macro-States in Chronic Myeloid Leukemia
Source: Cancer Res. Author manuscript; Available in PMC 2026 Oct 19. (PMC13679914; doi:10.1158/0008-5472.CAN-25-4371)
Supplement: Supplementary file 1 [file NIHMS2215895-supplement-Fig__S1.pdf]

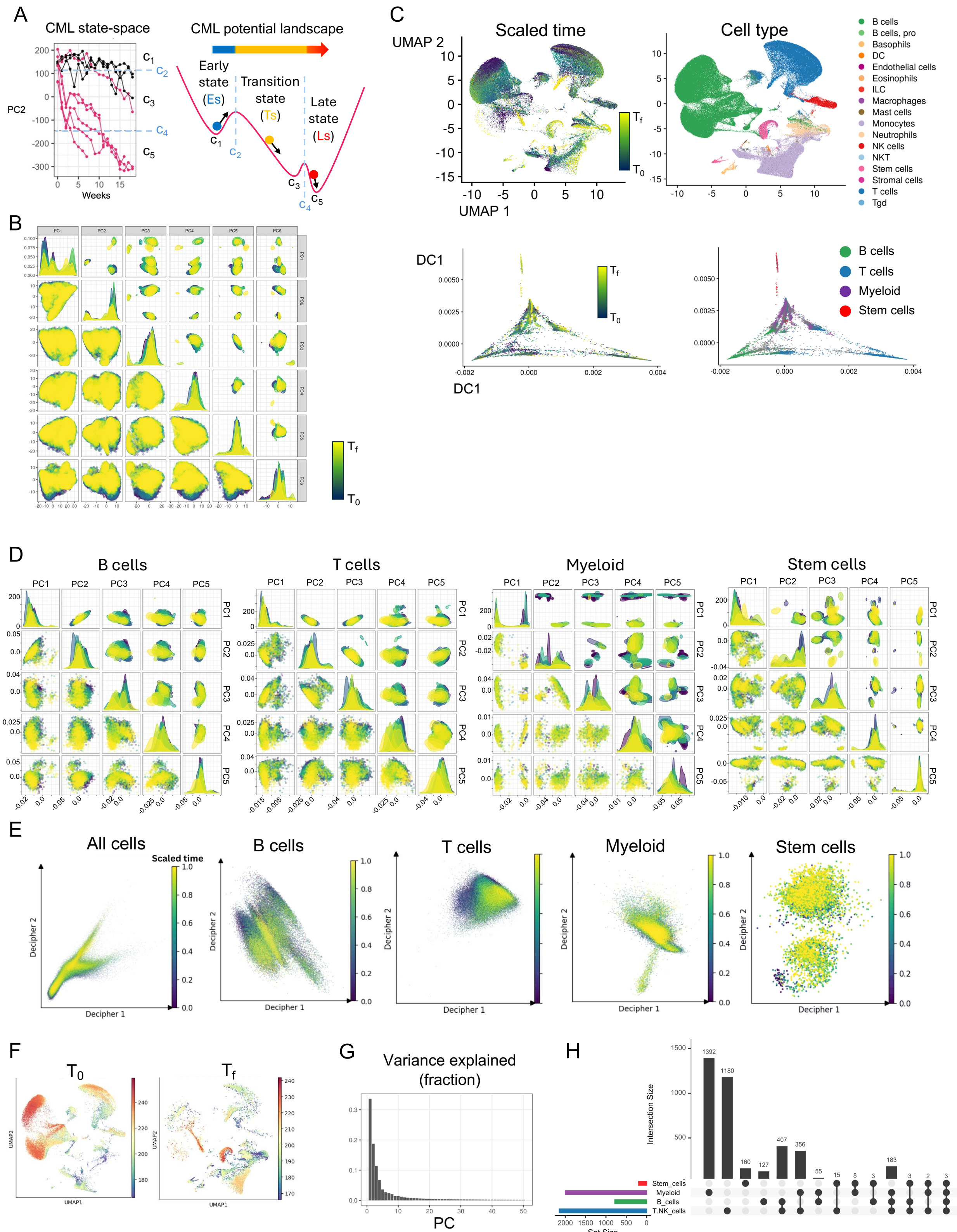

Figure S1: **Single cell analysis to identify CML state.**

A) Mouse trajectories in the CML state-space constructed from the time-series bulk RNA-seq data (*left*)[8]. See Frankhouser et al. 2024 for full details on state-space construction, critical point

identification, and potential landscape determination. The representative three-well potential describes how each mouse was modeled as undergoing Brownian motion in a potential energy landscape (*right*). The critical points of the potential were used to define the phenotypic disease states (Es, Ts, Ls). **B**) PCA was performed on the mean-centered expression of all genes using all cells from the time -series scRNA-seq data from the CP CML mice. For the mice that developed CML, they progressed at different rates so each mouse's week 0 (T_0) and their final time point (T_f) were scaled range between 0 and 1 so that their CML trajectories were aligned. The scaled time was used to try to identify a state-transition at the SC-level in PC1-5 where the elbow of the scree plot occurred (Fig. S1I). **C**) UMAP was also performed on all cells from all CP CML samples to try to identify a SC-level state-transitions using scaled time. Labeling each cell by the cell type shows that UMAP primarily separates different cell populations of the PBMCs. **D**) For each cell type category, SVD was performed on the mean-centered gene expression and PCs 1-5 were colored by the scaled time of the leukemic mice to identify a leukemia state-transition within cell type at the SC-level. **E**) The cell state density tool Mellon was used to try to identify SC-level leukemic state-transitions[9]. **F**) Decipher, a tool for constructing a latent space from control and perturbed cell populations to identify trajectories of derailed cells, was also used on all cells and on each cell type separately[9]. After building the Decipher space using the cells from the healthy time points vs the post induction time points, the resulting latent spaces were colored using the samples scaled time. **G**) Scree plot of the variance explained by the PCs that result from performing PCA on the mean-centered gene expression of the SC-level scRNA-seq data. **H**) Upset plot of the DEGs identified by comparing the cells from healthy (T_0) vs leukemic (T_f) time point samples from the leukemic mice for each cell type.
