# Supplementary material for: Longitudinal Single-Cell RNA Sequencing Reveals Evolution of Micro- and Macro-States in Chronic Myeloid Leukemia
Source: Cancer Res. Author manuscript; Available in PMC 2026 Oct 19. (PMC13679914; doi:10.1158/0008-5472.CAN-25-4371)
Supplement: Supplementary file 2 [file NIHMS2215895-supplement-Fig__S2.pdf]

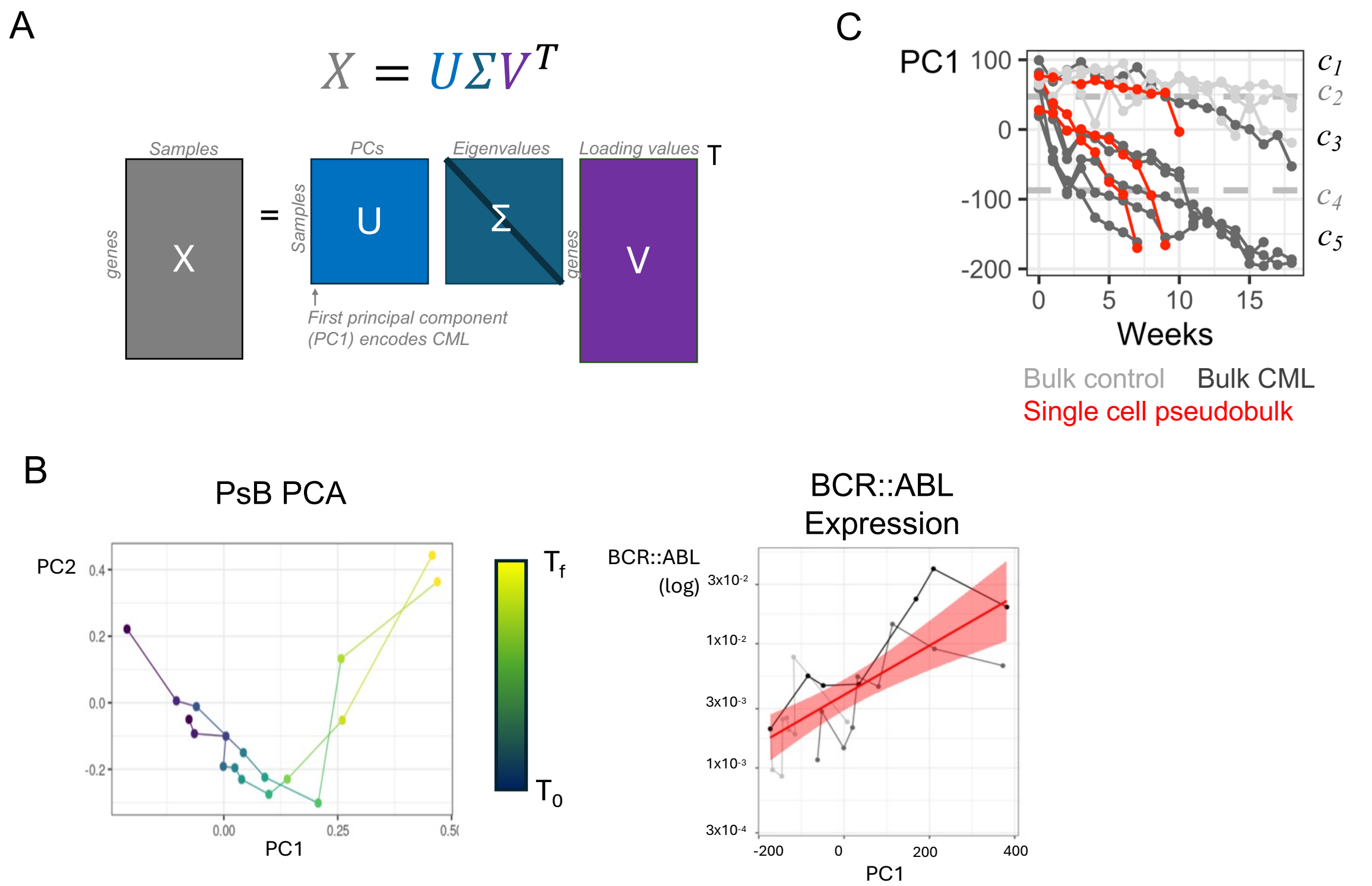

Figure S2: Construction of the CML state-space in PsB data

A) Singular value decomposition (SVD) equation and a representation of how it was applied to the PsB gene expression matrix. B) The mouse pseudobulk (PsB) trajectories are shown in PC1 vs PC2 (left). PC1 was identified as the PsB state-space as it was the only PC that showed concordance with the expression of the BCR::ABL leukemia oncogene (right). **C)** The PsB trajectories are compared to the bulk trajectories in the bulk CML state-space which was scaled to span PC1 from the PsB data.
