# Supplementary material for: Longitudinal Single-Cell RNA Sequencing Reveals Evolution of Micro- and Macro-States in Chronic Myeloid Leukemia
Source: Cancer Res. Author manuscript; Available in PMC 2026 Oct 19. (PMC13679914; doi:10.1158/0008-5472.CAN-25-4371)
Supplement: Supplementary file 6 [file NIHMS2215895-supplement-Fig__S6.pdf]

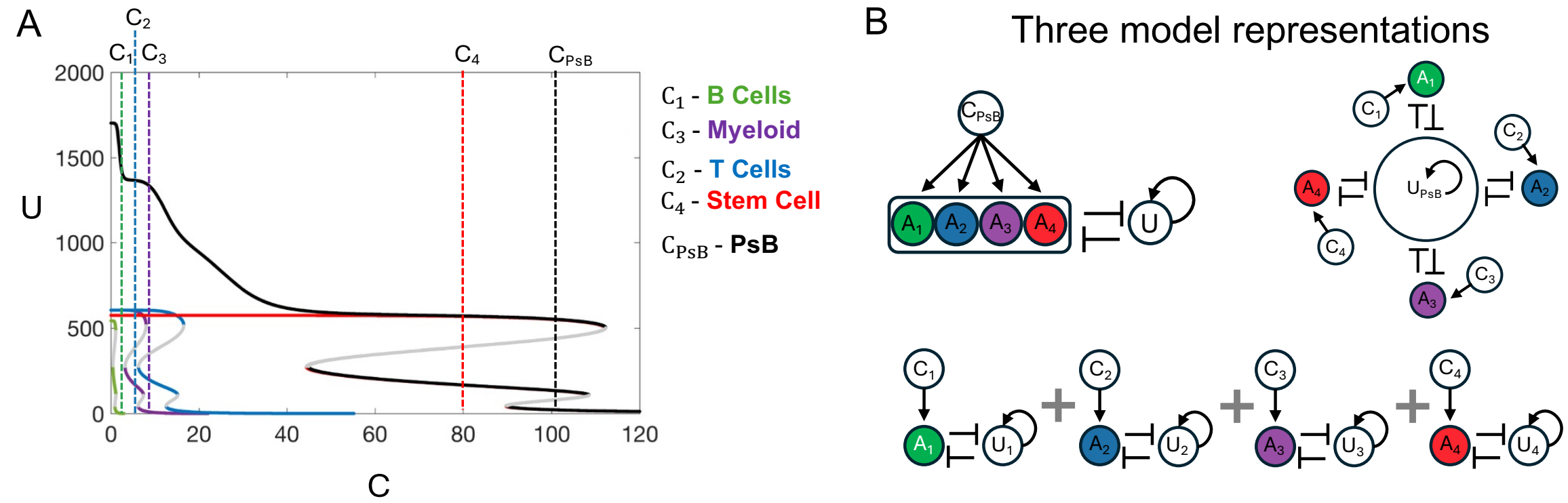

Figure S6: Model of CML dynamics from cell type transcriptomes
A) Bifurcation diagram that produces the ctPsB and PsB potentials shown in Figure 3B. The potentials are determined by setting different values of C_{ct}, the BCR::ABL signal levels, for each cell type in these bifurcations as $C_1 = 1; C_2 = 7; C_3 = 14; C_4 = 80$, which results in a PsB BCR::ABL signal of $C_{PsB} = 102$. **B**) Three representations of the model that related the ctPsB dynamics to the PsB dynamics.
