# Supplementary material for: Longitudinal Single-Cell RNA Sequencing Reveals Evolution of Micro- and Macro-States in Chronic Myeloid Leukemia
Source: Cancer Res. Author manuscript; Available in PMC 2026 Oct 19. (PMC13679914; doi:10.1158/0008-5472.CAN-25-4371)
Supplement: Supplementary file 7 [file NIHMS2215895-supplement-Fig__S7.pdf]

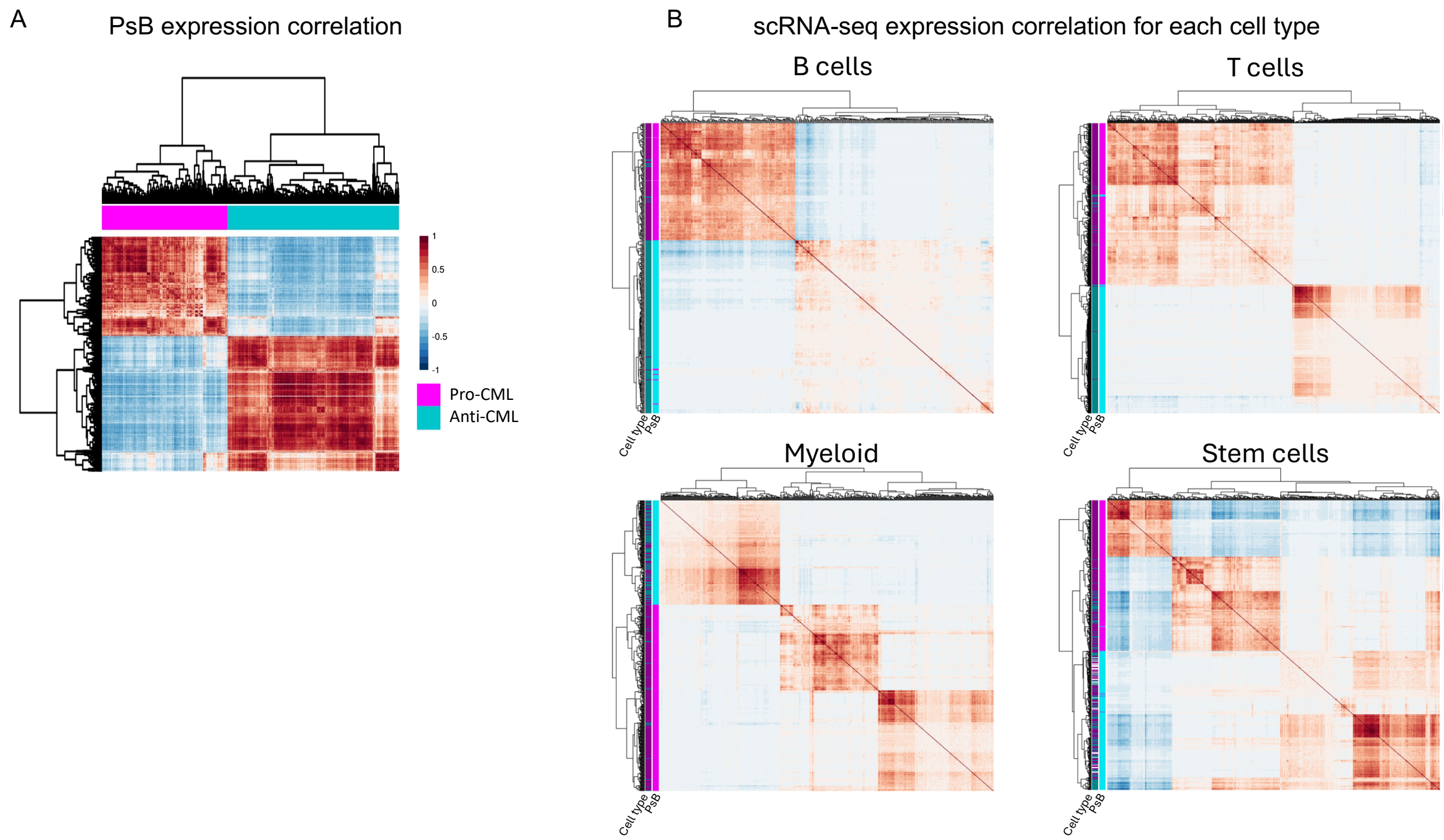

Figure S7: Characteristics of antagonistic teams of genes in PsB and sc-level data

A) The PsB expression for the top 90 percentile of eigengenes by magnitude were correlated for the CP CML mice. The gene-by-gene correlation matrix for these 2951 genes produces two sets of strongly anticorrelated genes. This correlation structure is similar to the antagonistic teams of genes observed in the expression of a gene network that has demonstrates phenotype switching.

B) The same gene-by-gene correlation matrix was produced using the scRNA-seq data for each cell type which did not show strongly anti-correlated sets of genes (data not shown). However, within each cell type, different subsets of those genes did produce two or more anti-correlated teams of genes (Table S6). The contribution to leukemia is shown for each gene based on the gene's contribution to both the PsB state-space and the ctPsB state-space for each corresponding cell type.
